# Predicting the Thermodynamic Limits of Metal–Organic Framework Metastability

**DOI:** 10.1021/jacs.5c20253

**Published:** 2026-05-11

**Authors:** Blake Dallmann, Aryan Saha, Andrew S. Rosen

**Affiliations:** 1 Department of Chemical and Biological Engineering, 6740 Princeton University, Princeton, New Jersey 08544, United States; 2 Department of Electrical and Computer Engineering, 6740 Princeton University, Princeton, New Jersey 08544, United States

## Abstract

The vast combinatorial space of metal–organic frameworks (MOFs) has led to their widespread consideration across diverse application areas. That said, much remains unknown about what factors govern their thermodynamic stability. Herein, we use density functional theory to compute the formation energy and construct convex hull phase diagrams for 20,000+ MOFs and coordination polymers. Using the energy above hull as a measure of stability with respect to decomposition and phase transitions, we validate and expand upon previous hypotheses that all MOFs are thermodynamically metastable and that there is an inherent energetic penalty associated with permanent porosity. We also describe how MOF composition and metal/linker identity influence the degree of metastability, in addition to demonstrating how the energy above hull can be used as a synthesizability metric for newly proposed MOFs. To democratize the knowledge gained from our study, we have released the QMOF-Thermo Database, which is the first database of energy above hull values for MOFs and coordination polymers. We conclude by using the QMOF-Thermo Database to benchmark the ability of pretrained machine learning interatomic potentials to predict the energy above hull of MOFs, and we identify opportunities to correct their performance.

## Graphical abstract

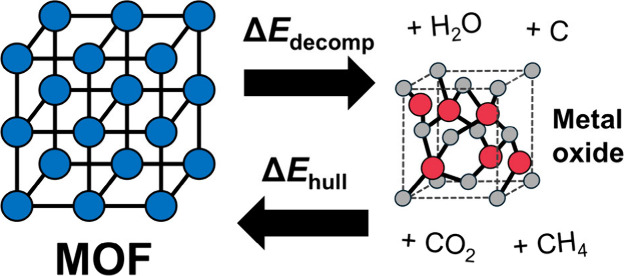

## Introduction

Over the past several decades, advances in materials chemistry have led to the discovery of atomically tunable, porous framework solids.^1^ The most popular subclass of such materials, metal–organic frameworks (MOFs), are composed of spatially isolated metal ions or inorganic clusters connected by organic ligands. To date, tens of thousands of MOFs have been synthesized,^2,3^ and virtually unlimited more can be proposed based on different combinations of inorganic and organic building blocks.

Given the vast combinatorial space of plausible structures, computational methods have emerged as an efficient means of identifying top-performing MOFs for a given application.^4–6^ However, there remains a large disconnect between promising MOFs designed on the computer and those that have been successfully realized in the laboratory setting, a challenge that is likely to grow with the advent of generative artificial intelligence (GenAI) methods for MOF design.^7^ More broadly, despite the enormous interest in MOFs from both computational and experimental scientists, much remains unknown about what factors govern MOF stability and what limits may exist with regards to their synthesizability.

There are numerous factors that collectively influence MOF stability, including but not limited to stability with respect to thermal, chemical, and mechanical stimuli. From a computational standpoint, several machine learning models have been developed to rapidly predict the temperature stability,^8^ solvent removal stability,^8^ water stability,^9,10^ and mechanical stability^11^ of a given MOF. Another closely connected research direction involves developing heuristics to assess the likelihood of a MOF being synthesizable. With this goal in mind, molecular dynamics simulations have been used to identify bounds of synthesizability based on the free energy required to switch a MOF from an artificial Einstein crystal to a force field representation.^12,13^ Furthermore, prior work has shown that the degree of linker strain within the MOF unit cell can be used as a metric for its likelihood of synthesizability.^14^

In the present work, we have chosen to focus on one of the most fundamental aspects of MOF chemistry: their thermodynamic stability, particularly with regard to decomposition. *Ab initio* methods based on density functional theory (DFT) have become a widely adopted means of predicting the thermochemistry of solid-state materials.^15^ In the MOF literature, numerous studies have been carried out where the DFT-predicted formation energy is used as a surrogate for thermodynamic stability,^16–23^ often with the implication that a negative formation energy from the constituent elements suggests the MOF is stable or synthetically viable. However, as we will demonstrate in this work, relying on predicted formation energies is not an ideal proxy for stability in practice. Furthermore, thermocalorimetry experiments have shown that several synthesized MOFs are known to have positive formation enthalpies from the constituent elements.^24^

Like any material, the thermodynamic stability of a MOF can be quantified by its stability with respect to phase transitions into other crystal structures at a fixed composition as well as decomposition into competing materials with the same average chemical composition as the material of interest.^15^ Determining thermodynamic stability with respect to phase transition is relatively straightforward: one can simply compute the relative energy between the various polymorphs and determine which structure has the lowest relative energy. This approach has been applied throughout the literature, most notably for the successful *ab initio* prediction of MOF crystal structures.^25,26^ Determining MOF stability with respect to decomposition can be a more complex task, however, as it requires knowledge about the formation energies of the hundreds or thousands of chemical compounds that a MOF could plausibly decompose into under equilibrium conditions.

To account for both phase transitions and decomposition, the convex hull formalism can be applied.^15,27^ From a convex hull phase diagram, the materials that lie on the convex hull are thermodynamically stable, whereas materials above the hull are thermodynamically unstable such that their decomposition or phase transition would further decrease the energy of the system. For instance, the computed energies of over 200,000 solid-state materials on the Materials Project have made it possible to construct convex hull phase diagrams that describe their thermodynamic stability,^28,29^ from which various design principles have been derived.^30,31^ Although the construction of convex hull diagrams has become a central component of computational materials science, such methods have yet to be applied to MOFs. The closest example in the literature is recent work by Sholl and co-workers, who describe the relative stability of different metal combinations in a high-entropy, lanthanide-based MOF using a convex hull-inspired approach built around the energy of mixing.^32^

From an experimental thermochemistry perspective, combustion calorimetry studies of several prototypical MOFs have been published to date,^33^ including but not limited to MOF-5, HKUST-1, MIL-53, and various zeolitic imidazolate frameworks (ZIFs).^24,34–41^ Based on these studies, it has been proposed that crystalline porous materials with empty pores are exclusively metastable with respect to their corresponding dense phases.^42^ Furthermore, the available calorimetric data suggests that (beyond some critical threshold) there exists little thermodynamic penalty for synthesizing MOFs with higher porosities.^37^ Given the relatively small sample size of MOF calorimetry data that has been published in the literature, the generality of these findings remain to be seen.

In this work, we construct the first convex hull phase diagrams for MOFs, from which we have calculated the thermodynamic stability of 20,000+ MOFs and structurally related coordination polymers. We conclude that the energy above the convex hull (Δ*E*_hull_), which is a thermodynamic quantity derived from the convex hull diagram that represents a MOF’s propensity to decompose into more stable materials, is a more reliable descriptor of MOF stability than formation energy from the elements (Δ*E*_form_). We also find that Δ*E*_hull_ can be used as a metric to reject unfeasible hypothetical MOF structures produced by topology-based or GenAI methods. With this data set of MOF Δ*E*_hull_ values, we support and further expand upon previous claims in the experimental literature that all MOFs are thermodynamically metastable and that there is an inherent thermodynamic penalty associated with increasing porosity, which plateaus for highly porous structures. We identify common thermodynamic stability trends beyond porosity as well, including the prevalence of hard Lewis acid metals bonded to hard Lewis base ligands in the more thermodynamically stable MOFs and an apparent minimum in Δ*E*_hull_ as a function of the carbon fraction in the MOF.

To democratize the knowledge gained from our high-throughput study, we have released the QMOF-Thermo Database,^43^ which contains the DFT-computed formation energies and energy above hulls for 20,373 MOFs and coordination polymers derived from the Quantum MOF (QMOF) Database.^44,45^ We conclude by using the QMOF-Thermo Database to carry out the first benchmark of foundation machine learning interatomic potentials (MLIPs) for predicting the Δ*E*_hull_ of MOFs. We find that the accuracy of foundation MLIPs to predict the Δ*E*_hull_ of MOFs is limited by the need to accurately model both MOFs and their chemically distinct decomposition products, although this can be corrected in a *post hoc* manner.

## Results and Discussion

### Investigating MOF Metastability with Δ*E*_hull_

To construct the QMOF-Thermo Database, we computed the formation energy for each of the 20,373 MOFs in the QMOF Database and their potential thermodynamic decomposition products, as described in Figure S1. This process required calculating the total energy of 7889 “reference structures” from the Materials Project at the same PBE-D3­(BJ) level of theory as that used to construct the original QMOF Database. This consistent set of formation energies for the MOFs and plausible decomposition products was then used to construct the convex hull diagrams, as outlined in Figures S2 and S3. Importantly, the convex hull does not describe the MOF synthesis process itself; as such, the convex hull should only be interpreted as describing the thermodynamic (meta)­stability of a MOF with respect to more stable decomposition products.

With the newly created QMOF-Thermo Database, we first sought to compare the formation energy from the elements (Δ*E*_form_) and energy above hull (Δ*E*_hull_) since this is the first time that the latter has been computed for MOFs. By way of example, the Δ*E*_form_ for MOF-5 with the formula Zn_4_O­(bdc)_3_ (bdc^2–^ = 1,4-benzene­dicarboxylate) is predicted to be –2496 kJ/(mol formula unit) (equivalent to –0.49 eV/atom) and corresponds to the formation reaction 24C + 6H_2_ + ^13^/_2_O_2_ + 4Zn → Zn_4_O­(bdc)_3_. Likewise, the Δ*E*_hull_ of MOF-5, which is the negative of the decomposition energy (Δ*E*_dec_), is predicted to be Δ*E*_hull_ = –Δ*E*_dec_ = 1041 kJ/(mol formula unit) (equivalent to 0.20 eV/atom). This energy difference corresponds to the thermodynamically favored decomposition reaction Zn_4_O­(bdc)_3_ → ^45^/_2_C + 6H_2_O + ^5^/_2_ZnO + ^3^/_2_ZnCO_3_ based on our convex hull phase diagram, which considers a closed system in the equilibrium limit. We note that the set of decomposition products are similar to those observed in calcination studies, which show that MOF-5 thermally decomposes into carbon, water, ZnO, and CO_2_.^46^ The fact that ZnCO_3_ is predicted instead of CO_2_ can be explained when accounting for finite temperature and pressure in the convex hull construction (Figure S6); at 1 bar pressure and temperatures above ∼320 K, ZnO and CO_2_ become the most stable products as opposed to ZnCO_3_ in our phase diagram construction.

The distributions of Δ*E*_form_ and Δ*E*_hull_ for MOFs categorized as synthesized or hypothetical are shown in Figure 1. Despite the frequent use of a negative Δ*E*_form_ as an indicator of synthetic feasibility in the literature on MOFs, the vast majority (91.8%) of hypothetical MOFs are predicted to have a negative Δ*E*_form_ (Figure 1A) even though it is unlikely that most of these MOFs are synthetically realizable. Additionally, an appreciable fraction (9.8%) of synthesized MOFs are predicted to have a positive Δ*E*_form_, which has also been reported in calorimetry experiments.^24^ From the results in Figure 1A, we can conclude that Δ*E*_form_ is not a sufficient metric to filter out synthetically inaccessible MOFs, as both the experimentally characterized and hypothetical MOFs have highly overlapping Δ*E*_form_ distributions despite being derived from notably different distributions of materials.^47^

In contrast, the distributions of Δ*E*_hull_ for synthesized and hypothetical MOFs are distinct from one another (Figure 1B), with the median Δ*E*_hull_ for the synthesized MOFs (0.21 eV/atom) being significantly lower than that of the hypothetical MOFs (0.29 eV/atom). This is to be expected, as many of the hypothetical MOFs can have combinations of nodes, linkers, and/or topologies that may not be energetically favorable. Furthermore, some hypothetical MOFs reach exceptionally large Δ*E*_hull_ values upward of 0.8 eV/atom, whereas the upper limits of Δ*E*_form_ are comparable for both the synthesized and hypothetical MOF subsets. We note that computing Δ*E*_form_ and Δ*E*_hull_ for randomly selected hypothetical MOFs from the chemically diverse ARC-MOF Database^48^ (Figure S30) results in similar distributions to those shown in Figure 1. Collectively, these observations lend credence to the fact that Δ*E*_hull_ should be used in place of Δ*E*_form_ to gauge the thermodynamic stability of a newly proposed MOF, which is a practice that has already been adopted for other classes of solid-state materials.^15^

**Figure 1:**
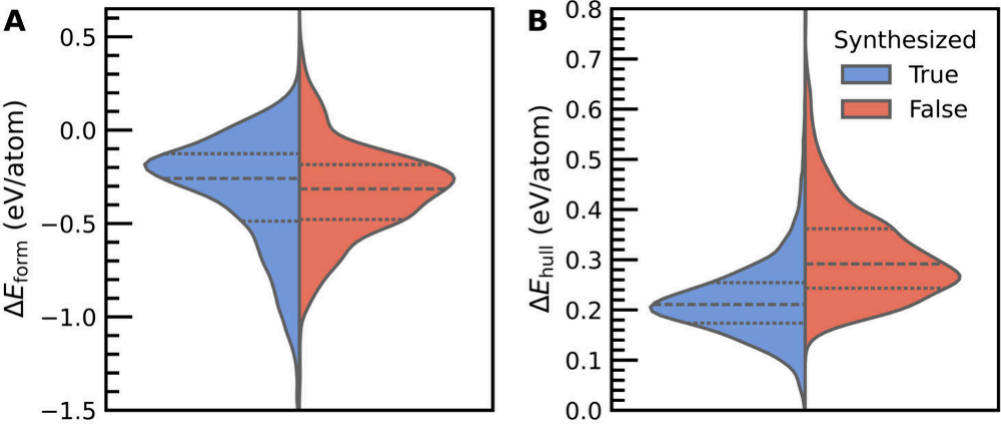
Violin plots of the DFT-computed (A) formation energy (Δ*E*_form_) and (B) energy above hull (Δ*E*_hull_) for synthesized (16,882) and hypothetical (3491) MOFs in the QMOF Database. The central dashed line represents the median, and the dotted lines represent the first quartile and third quartile in each distribution. Δ*E*_form_ is defined with respect to elemental phases. All energy values were computed at the PBE-D3­(BJ) level of theory.

For nearly all synthesized and hypothetical MOFs, Δ*E*_hull_ is predicted to be greater than zero (Figure 1B), indicating that they are thermodynamically metastable structures. These results support the hypothesis that virtually all crystalline MOFs are thermodynamically metastable, kinetically trapped phases.^42^ We emphasize that these results apply to idealized crystalline MOFs with evacuated pores, as amorphous or solvent-containing MOFs may or may not exhibit substantially different degrees of metastability. Despite being metastable, kinetic factors prevent known crystalline MOFs from decomposing into amorphous MOFs or other products.

Given the large data set size of MOF structures considered in the QMOF-Thermo Database, we provide strong evidence in support of previous MOF metastability claims that were based on thermocalorimetry experiments of ∼10 prototypical MOF structures.^33,37^ Out of the 20,373 MOFs that compose the QMOF-Thermo Database, only five (0.025%) MOFs appear on the convex hull (i.e., Δ*E*_hull_ = 0 eV/atom). Two of these anomalies (i.e., qmof-82d5634, qmof-40b4231) are nonporous coordination polymers composed of alkali metals and cyanurate linkers. The remaining three anomalies (i.e., qmof-50671ea, qmof-58c45b1, qmof-b5589d4) may be attributed to MOFs that contain elements with relatively few structures on the Materials Project (i.e., Tb, Tm, Pr). Identifying and computing the formation energies of all the thermodynamically relevant compounds that exist across 834 unique chemical spaces is a challenging (if not impossible) task; therefore, the calculated Δ*E*_hull_ values for the MOFs presented in this work should be thought of a lower bound, as including decomposition products of greater stability would only serve to lower the convex hull and increase the predicted Δ*E*_hull_. As such, the few MOFs with Δ*E*_hull_ = 0 eV/atom may, in fact, be metastable if additional structures in their chemical spaces were considered.

To better understand the relationship between Δ*E*_form_ and Δ*E*_hull_, we investigated the correlation and distributions of both quantities for MOFs in the QMOF Database and Materials Project crystal structures (Figure 2). Despite the fact that Δ*E*_hull_ is directly calculated from a collection of Δ*E*_form_ values, there is little correlation between Δ*E*_form_ and Δ*E*_hull_ for MOFs (Figure 2A); therefore, Δ*E*_form_ is not a suitable proxy for thermodynamic stability. The poor correlation between Δ*E*_form_ and Δ*E*_hull_ has already been discussed in the context of inorganic solid-state materials like those found on the Materials Project,^15^ which is evident upon inspecting the results in Figure 2B. The convex hull represents a material’s thermodynamically competitive chemical space, which extends beyond the simple picture of stability with respect to the elemental references. In comparing Figure 2A and Figure 2B, it also becomes clear that the thermodynamic stability of MOFs differs drastically from the solid-state materials found on the Materials Project. Most materials on the Materials Project reside relatively close to (or directly on) the convex hull, and a Δ*E*_hull_ of ∼0.2 eV/atom is considered rare even though it is quite common among MOFs. As a point of reference, while a Δ*E*_hull_ of ∼0.2 eV/atom is commonly considered to be high, it is not unprecedented; this value is comparable to the Δ*E*_hull_ of several known, metastable nitrides.^31^ For additional context, the explosive coordination polymer Pb­(N_3_)_2_ is predicted to have an energy above hull of 0.42 eV/atom according to the Materials Project.^29^

**Figure 2:**
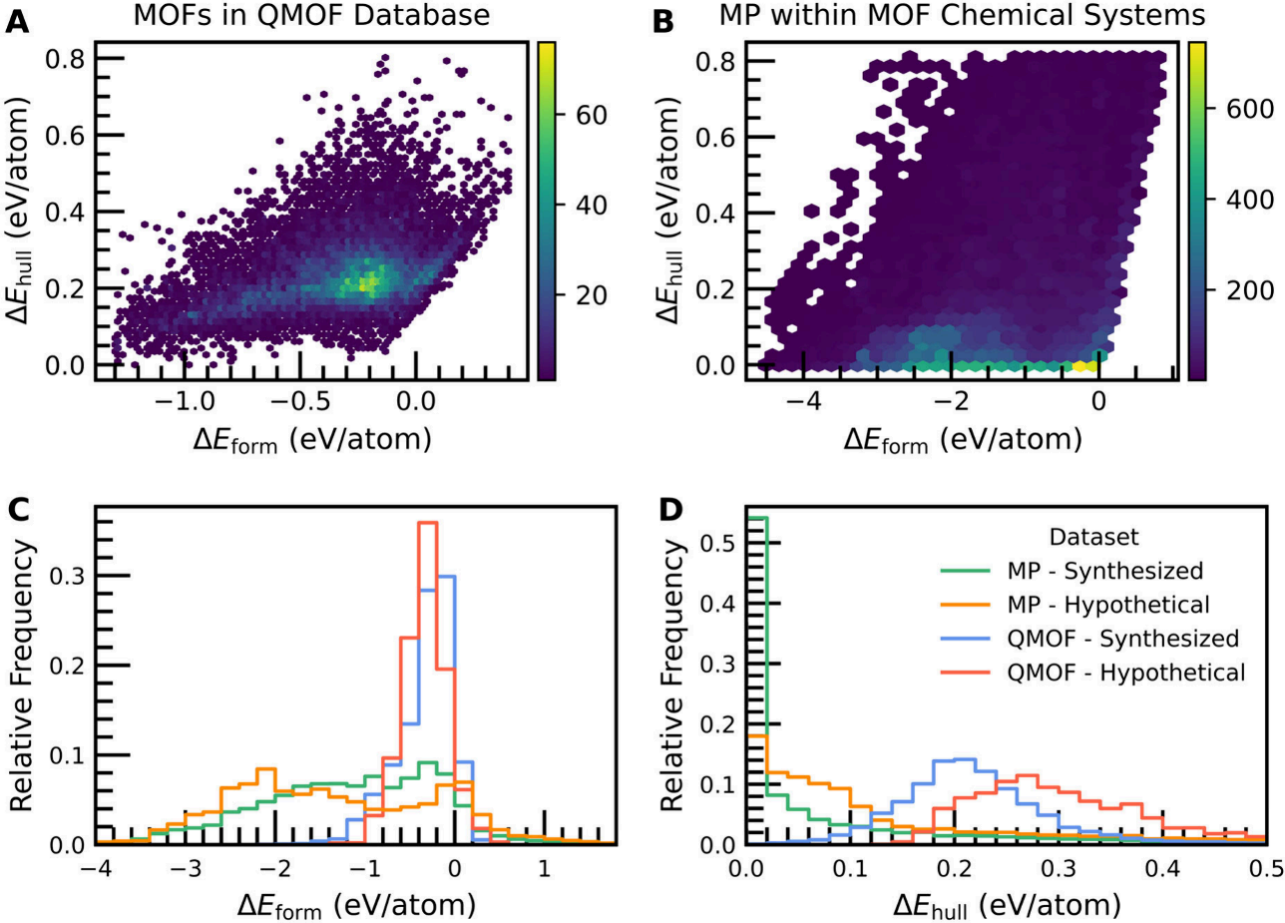
Formation energy (Δ*E*_form_) plotted against energy above hull (Δ*E*_hull_) for (A) MOFs in the QMOF Database and (B) Materials Project (MP) structures contained within at least one of the 834 unique chemical spaces in QMOF. The color bar shows the number of entries in each hexagonal bin. Histograms of (C) Δ*E*_form_ and (D) Δ*E*_hull_ for all synthesized (16,882)/hypothetical (3491) QMOF structures and synthesized (13,782)/hypothetical (18,916) Materials Project structures contained within at least one QMOF chemical space. The ranges of Δ*E*_form_ in (C) and Δ*E*_hull_ in (D) have been truncated to [−4.0, 1.8] and [0.0, 0.5] (eV/atom), respectively, for ease of visualization.

Compared to the solid-state materials on the Materials Project, the distribution of Δ*E*_form_ for the MOFs is shifted toward less exothermic values on average and takes on a substantially narrower range of values (Figure 2C), which can be attributed to the significant influence of carbon on Δ*E*_form_ (discussed further with Figure 7) and the narrower chemical space of the QMOF Database, respectively. When examining the Δ*E*_hull_ distributions for structures obtained from the Materials Project (Figure 2D), synthesized structures have a sharp peak close to the hull, and hypothetical structures have a right-skewed distribution with a median of 0.1 eV/atom. In contrast, the MOF distributions have less overlap between the synthesized and hypothetical materials based on Δ*E*_hull_, and the medians of both distributions are well above Δ*E*_hull_ = 0 eV/atom. Even though larger Δ*E*_hull_ values tend to be associated with a reduced likelihood of synthesizability,^30,31^ the inherent metastability of MOFs shifts their Δ*E*_hull_ distributions further away from the hull.

### Δ*E*_hull_ as a Synthesizability Metric

Given that Δ*E*_hull_ is a reliable quantifier of thermodynamic stability, a natural question arises: what value of Δ*E*_hull_ would render a MOF thermodynamically unfeasible? The large Δ*E*_hull_ distribution of 16,882 synthesized MOFs (Figure S9) sets a clear precedent for the physical bounds of Δ*E*_hull_ values across MOF chemical space. This distribution has a relatively large peak at 0.21 eV/atom and is slightly right-skewed, which suggests that although large values of Δ*E*_hull_ are thermodynamically accessible, there is a thermodynamic limit of Δ*E*_hull_ above which synthesizability is statistically unlikely. Although this argument for an upper limit in Δ*E*_hull_ is largely empirical, we can rationalize that an upper limit should exist; if the Δ*E*_hull_ is sufficiently large, the driving force toward the decomposition products would be equally large and at some threshold would make the MOF unlikely to be realizable. While the Δ*E*_hull_ distribution deviates from perfect normality (Figure S10), the large data set size justifies it to be representative of the entire population of synthesized MOFs, allowing empirical quantiles to provide reliable Δ*E*_hull_ thresholds in a manner similar to that done for inorganic materials.^31^

Figure 3 shows the empirical quantiles of Δ*E*_hull_ values for the synthesized MOFs in QMOF. These empirical quantiles provide potential Δ*E*_hull_ thresholds for identifying MOFs that are likely unsynthesizable (see Table S1 for specific threshold values). For example, 95% of synthesized MOFs in the QMOF database have a Δ*E*_hull_ below 0.353 eV/atom, which suggests this is a reasonable synthesizability threshold for newly proposed MOFs. More broadly, Δ*E*_hull_ can serve as an adjustable threshold to discard synthetically unfeasible MOFs in computational MOF design studies. As a further test, we searched all hypothetical MOFs in the QMOF Database for any that have been coincidentally synthesized. By comparing the MOFids^49^ listed in the QMOF Database with those listed in the CoRE MOF Database[Bibr ref3] (2025 v1.0), we found six instances of MOFs listed as hypothetical that have in fact been synthesized (Table S2). Without exception, all six MOFs fall below the 0.353 eV/atom Δ*E*_hull_ threshold.

**Figure 3:**
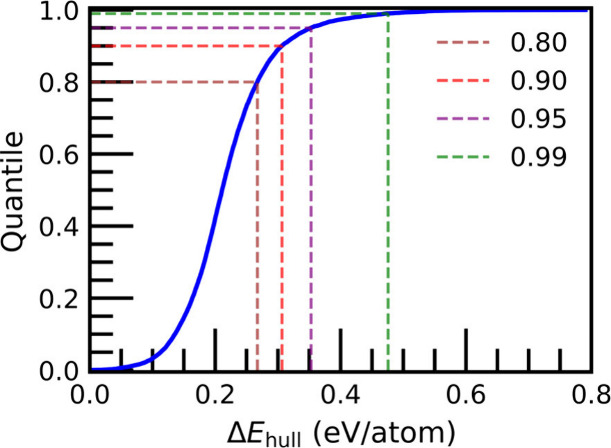
Quantile plot of Δ*E*_hull_ values for synthesized MOFs in the QMOF Database. The dashed guiding lines are for connecting a specific quantile to a value of Δ*E*_hull_.

To demonstrate the utility of the Δ*E*_hull_ threshold, the realized and hypothetical source data sets in the QMOF Database were analyzed by finding the proportion of MOFs (in their local minimum energy configuration) that are above the 0.353 eV/atom threshold (Table 1). Based on this analysis, data sets that rely on topology-based algorithms for MOF construction such as ToBasCCo^50^ (i.e., BoydWoo^51^), ToBaCCo^52,53^ (i.e., Anderson-Zr/Hf,^54^ ToBaCCo-Cu^53^) have a smaller proportion of thermodynamically feasible MOFs compared to the Mail-Order MOF-5 ^55^ (MO MOF-5) and hypothetical MOF-74 ^56^ (hMOF-74) databases. The latter set of databases contain hypothetical structures that are variants on a known MOF archetype, whereas the former set of databases used less restricted algorithms designed to produce novel chemistries and topologies. We hypothesize that these differences largely explain the data set dependent Δ*E*_hull_ distributions shown in Figure 4.

**Table 1:** Proportion of MOFs in Each QMOF Source Database with a Δ*E*_hull_ Larger Than the 0.353 eV/atom Δ*E*_hull_ Threshold^a^

	experimental MOFs			hypothetical MOFs						GenAI MOFs		
database	pyrene	CSD	CoRE	hMOF-74	MO MOF-5	GMOF	BoydWoo	Anderson-Zr/Hf	ToBaCCo-Cu	GHP	MOFFUSION	MOFDiff
fraction above threshold	0.00	0.05	0.04	0.02	0.02	0.14	0.38	0.41	0.53	0.00	0.15	0.4

a The Δ*E*_hull_ threshold is the value that 95% of synthesized MOFs in the QMOF Database do not exceed.

**Figure 4:**
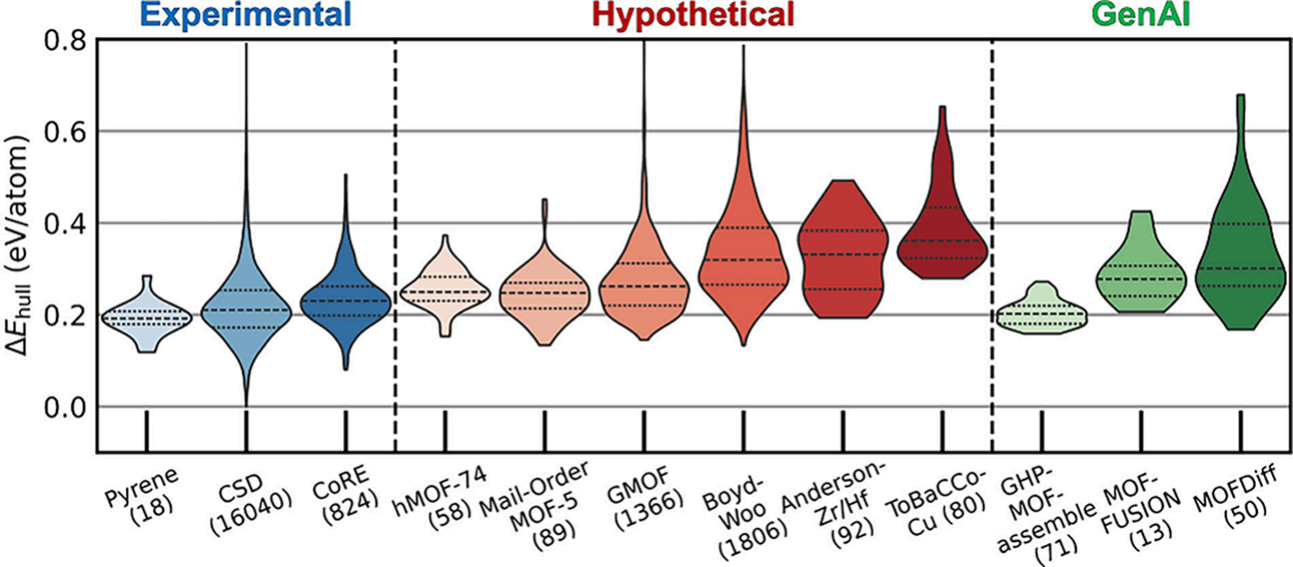
Violin plots of the Δ*E*_hull_ for MOFs in the QMOF Database sorted by source database and AI-generated MOFs sorted by model. The total number of MOFs corresponding to a source or model is given in parentheses. MOFs from synthesized databases are in shades of blue (left), MOFs from hypothetical databases are in shades of red (middle), and MOFs from generative AI models are in shades of green (right). The central dashed line in each violin represents the median, and the dotted lines represent the first quartile and third quartile in each distribution.

As is generally the case when structure relaxations are carried out, it can only be guaranteed that the MOFs are at a local minimum in the potential energy surface. As a result, our analysis is based on the thermodynamic stability of hypothetical MOFs in configurations representative of those produced by the underlying MOF construction algorithm, even if lower-energy configurations may exist. This is consistent with how hypothetical MOF data sets are generally used in practice; at the same time, it is also important to keep in mind that the large number of atoms in most MOF unit cells makes the predicted Δ*E*_hull_ values only weakly sensitive to configurational differences.

The results in Table 1 and Figure 4 demonstrate that Δ*E*_hull_ can be a valuable metric for discarding unfeasible MOFs in high-throughput studies focused on novel MOF design and discovery. Prior work indicates that the QMOF Database has an exceptionally small number of erroneous structures compared to other “computation-ready” MOF databases;^57^ however, with 20,000+ materials, there will inevitably be at least some structures that are worth removing. With this in mind, we sorted the MOFs in the QMOF Database by Δ*E*_hull_ and identified that qmof-42b55bb (CSD refcode = BIFSUF) had an exceptionally high Δ*E*_hull_ of 0.61 eV/atom that can be attributed to its ligands completely rearranging during the course of the DFT relaxation, and we subsequently removed it from the database; the validation checks in MOFChecker^58^ do not identify any issues with the structure. We note that Δ*E*_hull_ is not intended to be a replacement for existing MOF validation checkers^58–60^ or other simulation-derived synthesizability metrics, such as those based on free energy simulations^13^ or linker strain.^14^ Nonetheless, given the physical significance of Δ*E*_hull_ and its widespread use when describing other classes of solid-state materials, we consider it to be a complementary metric of interest.

The 95% Δ*E*_hull_ threshold was also used to test the thermodynamic stability of C–H–O–Zn MOFs constructed from GenAI models, following structure relaxation with DFT. The GHP-MOFassemble^61^ model has the lowest proportion of MOFs above the 95% Δ*E*_hull_ threshold (Table 1) and has a thermodynamically plausible Δ*E*_hull_ distribution centered around 0.20 eV/atom (Figure 4). The GHP-MOFassemble model generates novel MOF linkers in a fixed **pcu** topology with one of three metal nodes (i.e., Cu paddlewheel, Zn paddlewheel, Zn_4_O motif). Although this likely avoids thermodynamically unfavorable combinations of topology and metal nodes, GHP-MOFassemble is inherently more limited in terms of chemical diversity as a result. In contrast, MOFFUSION^62^ and MOFDiff^63^ are designed to generate novel linker, metal node, and topology combinations; for these GenAI models, the proportion of MOFs above the Δ*E*_hull_ threshold is similar to the hypothetical MOFs constructed using conventional topology-based algorithms (Table 1). MOFDiff is trained on structures from the BoydWoo data set of hypothetical MOFs,^63^ which may explain the similarities of the Δ*E*_hull_ distributions for MOFDiff and BoydWoo shown in Figure 4. As described further in the supplemental methods section in the Supporting Information, the GenAI MOFs were prefiltered by using the MOFChecker^58^ code to remove clearly erroneous structures. It must be emphasized that this process likely removes MOFs that would have particularly high Δ*E*_hull_ values; nonetheless, we chose to adopt a MOFChecker prefiltering step because it is representative of how these GenAI models are often used in practice and because the DFT calculations would be difficult to converge for especially problematic structures.

### Identifying MOF Structure–Stability Trends

In this subsection, we focus on using the DFT-computed thermochemistry to correlate the metastability of MOFs with their structural and chemical properties. In previous thermocalorimetry studies of 11 MOFs by Navrotsky and co-workers, the enthalpy with respect to the dense phase was plotted against normalized pore volume to make the following claims: (1) there is an inherent thermodynamic penalty associated with porosity; (2) this thermodynamic penalty for porosity plateaus as pore volume increases.^37^ We sought to use the large size of the QMOF-Thermo Database to gain further insight into the possibility of a thermodynamic penalty associated with permanent porosity and evaluate the hypotheses laid out by Navrotsky and co-workers, as demonstrated in Figure 5.

**Figure 5:**
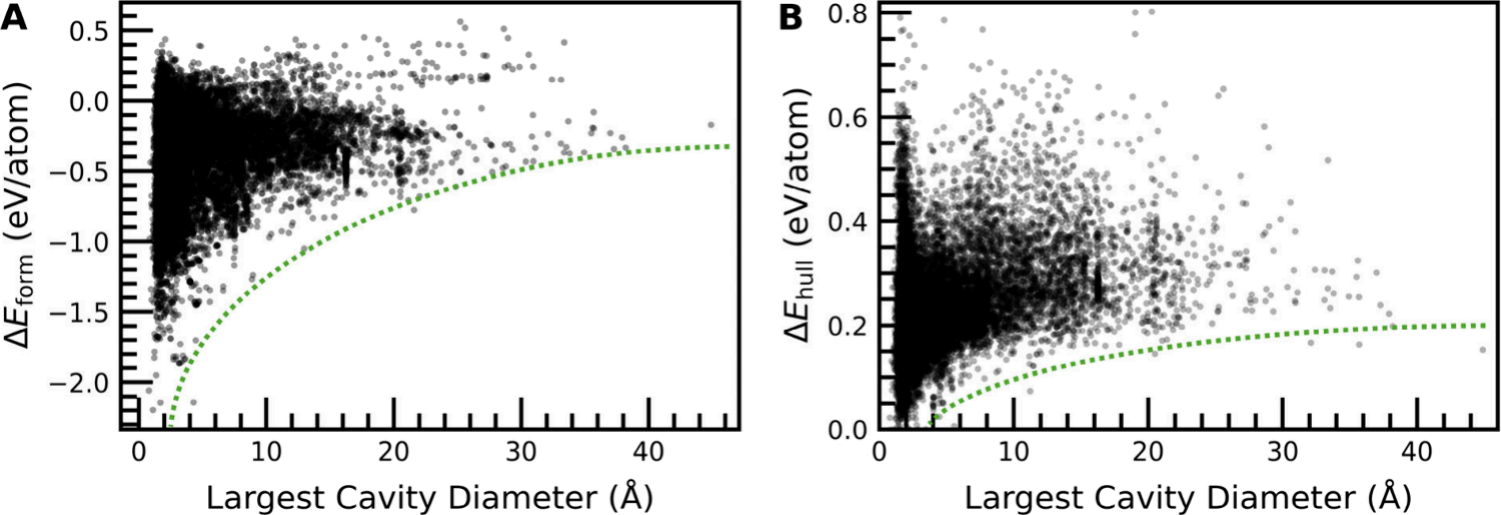
(A) Δ*E*_form_ and (B) Δ*E*_hull_ as a function of largest cavity diameter for synthesized and hypothetical MOFs in the QMOF Database. The dotted green lines are a guide for the eye.

Examining the distributions of Δ*E*_form_ and Δ*E*_hull_ for MOFs as a function of largest cavity diameter (LCD) in Figure 5, both distributions exhibit the same lower limit in energy that plateaus as LCD increases, which supports the claim that increasing porosity comes with an inherent, yet limited, thermodynamic penalty. Additionally, the fact that the lower limit in Δ*E*_hull_ eventually plateaus as a function of LCD in Figure 5B indicates that there are no inherent thermodynamic considerations that prevent the synthesis of ultraporous MOFs, which is in agreement with conclusions from Navrotsky and co-workers that were largely based on two high-porosity MOFs (MOF-177 and UMCM-1).^37^

In Figure 5A, the synthesized and hypothetical MOFs also exhibit a lower limit of Δ*E*_form_ that plateaus as LCD increases. Therefore, both Δ*E*_hull_ and Δ*E*_form_ contain an inherent, yet limited, thermodynamic penalty associated with porosity. This suggests that hypothetical MOFs in the QMOF database having a larger porosity on average than synthesized MOFs does not explain the difference in Δ*E*_hull_ and Δ*E*_form_ as a thermodynamic stability metric (Figure 1), since both Δ*E*_hull_ and Δ*E*_form_ scale with porosity.

Importantly, our results in Figure S15 indicate that many synthesized, low-porosity MOFs can still have large values of Δ*E*_hull_ (e.g., Δ*E*_hull_ > 0.35 eV/atom), which suggests that other structural and chemical properties (e.g., chemical bond strength, linker strain, composition) contribute significantly to the degree of thermodynamic metastability for these materials. In contrast with the trend observed by Navrotsky and co-workers that suggests the degree of metastability converges to a single value for ultraporous MOFs, we find a greater variation in metastability among ultraporous MOFs, except at very high porosity (i.e., LCD > 25 Å) where small sample sizes limit clear conclusions. Other metrics for porosity, such as pore-limiting diameter or probe-accessible volume, do not change these observations (Figure S16).

The few materials with both permanent porosity and a relatively low Δ*E*_hull_ (e.g., Δ*E*_hull_ < 0.075 eV/atom) that seem to cross the lower limit of Δ*E*_hull_ are not true MOFs. Closer inspection reveals that they are better classified as porous aluminophosphates (i.e., AlPOs) or materials with linkers that do not contain hydrogen (e.g., oxalates) (Figure S17). Although the presence of a lower limit in Δ*E*_hull_ as a function of porosity is apparent, it is less clear why an upper-limit appears to form as porosity increases for the synthesized MOFs (Figure S15). This may be due to factors beyond the thermodynamics itself, such as a difficulty in stabilizing the kinetically trapped, porous MOF phase when Δ*E*_hull_ is large or simply a matter of sampling bias in the QMOF Database.

We next turn our attention to how the node and linker chemistry may influence Δ*E*_hull_. A summary of MOF Δ*E*_hull_ trends across different node and linker properties is given in Figure 6. For the sake of simplicity, all Δ*E*_hull_ values in Figure 6 are for synthesized, monometallic MOFs in the QMOF Database. Figure 6A displays the median Δ*E*_hull_ of monometallic MOFs as a heat map on the periodic table. The median Δ*E*_hull_ varies significantly as a function of the metal identity; MOFs with group 3–6 metals tend to be more thermodynamically stable than MOFs with group 8–11 metals (except Au). Early transition metals in low valence states are known to exhibit relatively strong metal–ligand bonds due to the filling of their unoccupied d-orbitals.^64,65^ In contrast, late transition metals in high valence states will be more likely to exhibit strong π-backbonding, leading to weaker metal–ligand bonds.^66^ This difference in bond strength may contribute significantly to the difference in median Δ*E*_hull_ for the early and late transition metal-containing MOFs. The MOFs composed of group 12 metals, which have fully occupied d orbitals, tend to have a similar thermodynamic stability as the early transition metals.

**Figure 6:**
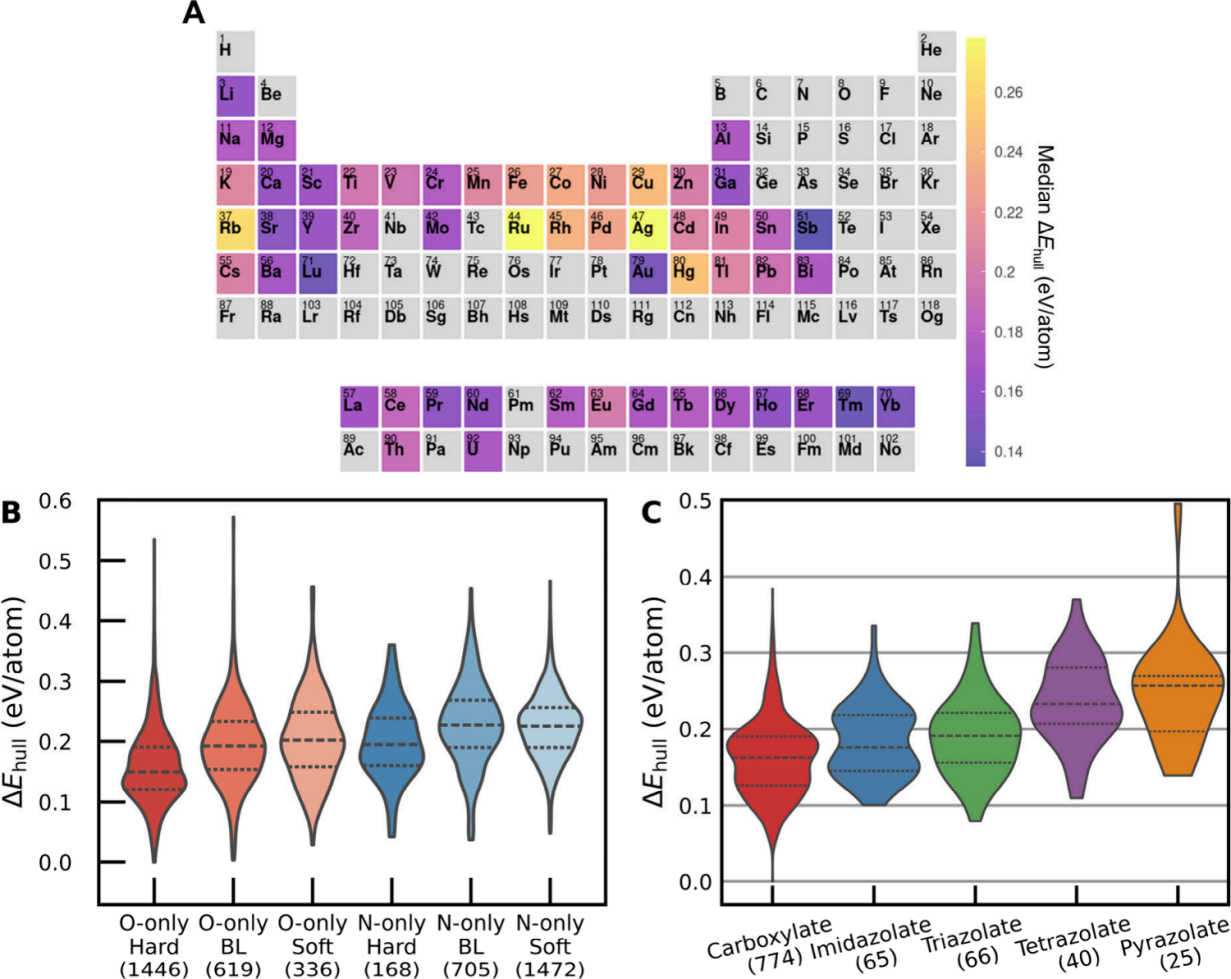
(A) Median Δ*E*_hull_ for 15,323 synthesized MOFs in the QMOF Database organized by metal. Structures with multiple metal elements or with less than eight entries in the QMOF Database are excluded. (B) Violin plots of the Δ*E*_hull_ for 4746 synthesized, monometallic MOFs in the QMOF Database organized by linker type and metal HSAB classification. BL denotes metals that are borderline Lewis acids. For the O-only and N-only linker types, only M–C–H–O and M–C–H–N (M = metal) chemical spaces were included, respectively. Multimetallic MOFs are excluded. A more detailed distribution of MOFs categorized by metal cation can be found in Figure S19. (C) Violin plots of the Δ*E*_hull_ for 970 synthesized MOFs in the QMOF Database organized by the type of linker. For the carboxylate and nitrogen-containing linkers, only M–C–H–O and M–C–H–N chemical spaces were included, respectively. MOFs with multiple types of linkers were excluded. For the labels in (B) and (C), the number in paratheses is the number of MOFs in that distribution. The central dashed line in each violin represents the median, and the dotted lines represent the first quartile and third quartile in each distribution.

A related perspective to evaluate metal–ligand bond stability is through hard–soft acid–base theory (HSAB), which predicts that hard Lewis acids (i.e., metals with high oxidation state, small ionic radii) will form strong bonds with hard Lewis bases, such as carboxylates in MOFs. Soft Lewis acids (i.e., metals with low oxidation state, large ionic radii) will form strong bonds with soft Lewis bases, such as azolates in MOFs.^67–70^ Figure 6B shows the distributions of Δ*E*_hull_ for monometallic MOFs grouped by linker type (i.e., O-containing or N-containing) and metal HSAB classification. MOF structures with hard metals binding to hard O-containing (i.e., carboxylate) ligands exhibit the lowest median of Δ*E*_hull_ values (median Δ*E*_hull_ = 0.15 eV/atom) relative to the other groupings, which we hypothesize is a hard–hard pair that enables the formation of strong chemical bonds. Notably, other O-containing groupings have a median Δ*E*_hull_ > 0.19 eV/atom, which are hard–borderline or hard–soft pairs that are less favorable. Examining the MOF distributions with N-containing linkers, all such metal–ligand pairings have a median Δ*E*_hull_ > 0.19 eV/atom. Unlike the MOFs with O-containing linkers that are dominated by carboxylates, the N-containing linkers are substantially more diverse. Whereas azolates and cyanides are considered soft Lewis bases, pyridines and azides are more borderline Lewis bases, and many amines are hard Lewis bases.^70,71^ Since nitrogen-containing ligands are generally more diverse in hardness/softness depending on the chemical environment, we hypothesize that this makes less stable bonds (i.e., soft–hard pairs) more prevalent, which increases the median Δ*E*_hull_ for these MOFs.

To help deconvolute the Δ*E*_hull_ trends as a function of linker chemistry, we have plotted the Δ*E*_hull_ distributions for carboxylate and azolate-containing MOFs in Figure 6C. Overall, MOFs that contain only carboxylate linkers tend to be more thermodynamically stable than MOFs that contain only azolate linkers. One plausible explanation for this observation is that the carboxylate MOFs in the QMOF Database are primarily composed of hard metals (e.g., Li^+^, Ca^2+^, Mg^2+^, Mn^2+^, lanthanides) that bind favorably with the hard carboxylate groups, whereas the azolate MOFs have a larger proportion of borderline metals (e.g., Co^2+^, Cu^2+^) that lead to weaker bonds with the soft azolate groups based on HSAB theory (Figure S20). If only MOFs composed of late transition or post-transition metals (e.g., Zn^2+^, Cd^2+^, Ag^+^) are included in determining the median Δ*E*_hull_ as a function of linker type, the difference in stability between the azolate and carboxylate MOFs is significantly reduced (Figure S21).

Although we have primarily used Δ*E*_hull_ as a quantitative metric for MOF thermodynamic stability, it is equally important to recognize that Δ*E*_hull_ can be used to identify the stable phases that each MOF may decompose into under equilibrium conditions. The most common MOF decomposition products derived from our convex hull phase diagram are shown in Figure 7A. The predicted decomposition products are in qualitative agreement with many of the classes of compounds that are produced in the irreversible, high temperature treatment of MOFs like that obtained via calcination or thermal gravimetric analysis. For instance, the formation of carbon, H_2_O, CH_4_, CO_2_, and N_2_ are all to be expected on the basis of prior experimental studies.^72,73^ Metal oxides of varying stoichiometries are also reasonably predicted to be major decomposition products (refer to Figure S22 for a histogram of the metal-containing decomposition products). Several of the other most common decomposition products involve ammonia or ammonium salts, although the precise entries that appear in Figure 7A may be susceptible to relatively small changes in the predicted formation energies. All this being said, it is important to re-emphasize that the convex hull diagrams in this work describe a closed system in the equilibrium limit, and the exact set of decomposition products for a given MOF may differ from what would be observed under common experimental conditions. For instance, one would typically expect species like NH_3_·H_2_O to phase separate into NH_3_ and H_2_O, both of which are already common decomposition products as shown in Figure 7A.

As shown in Figure 7A, the vast majority (i.e., 20,180) of the MOFs have carbon as one of its decomposition products, which indicates there is a significant driving force toward decomposition to elemental carbon (i.e., carbon is a thermodynamic sink) under the closed, equilibrium conditions considered in the convex hull approach. Given the large driving force for decomposition toward elemental carbon, as the carbon fraction in the MOF increases, so does the Δ*E*_form_ (Figure 7B) and Δ*E*_hull_ (Figure 7C) on average. The positive correlation in Δ*E*_form_ and carbon fraction is observed for all materials, not just MOFs, which results in the majority of materials on the convex hull having a low carbon fraction (Figure S23). A correlation in the Δ*E*_hull_ for MOFs was not observed for any other common element, although some correlations in oxygen, nitrogen, and hydrogen were observed for Δ*E*_form_ (Figure S24).

Notably, there is an apparent minimum in the possible Δ*E*_form_ and Δ*E*_hull_ values as a function of the carbon fraction. For instance, no MOFs with a carbon fraction of 0.5 have Δ*E*_form_ < –1.0 eV/atom or Δ*E*_hull_ < 0.1 eV/atom even though there are many such MOFs with a carbon fraction of 0.2. A minimum in Δ*E*_hull_ was not observed for any other common elements (Figure S24). This suggests that there tends to be an increasing energy penalty in MOFs with increasing compositions of carbon. We note that the increase in Δ*E*_form_ and Δ*E*_hull_ as a function of carbon fraction (and the presence of stability-based lower bounds) cannot be attributed solely to an energy penalty associated with increasing porosity, as many low-porosity MOFs and coordination polymers exist for nearly all carbon fractions (Figure S25).

**Figure 7:**
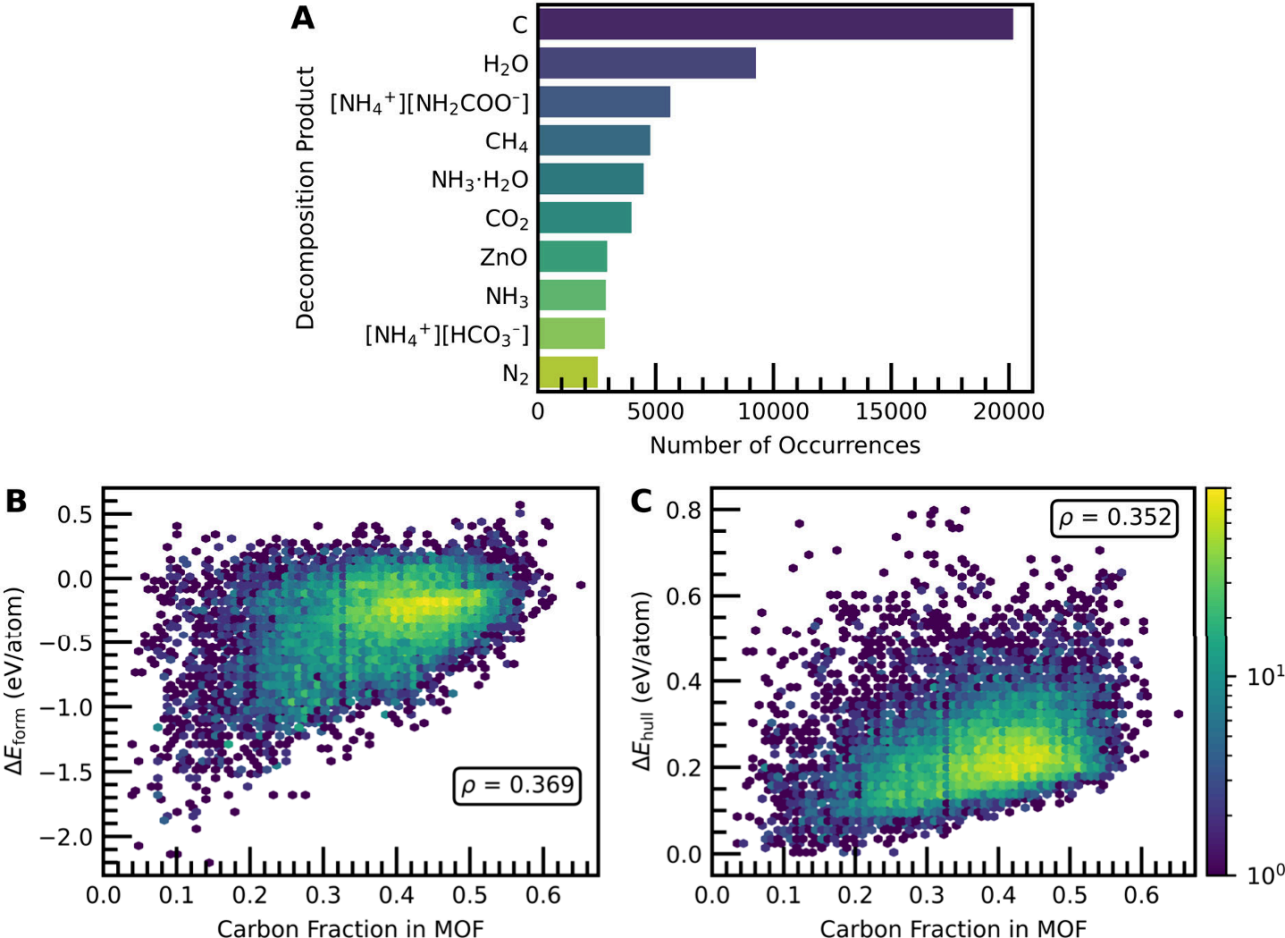
(A) Number of occurrences for the ten most frequent MOF decomposition products. (B) Δ*E*_form_ and (C) Δ*E*_hull_ as a function of the stoichiometric carbon fraction, *x*_C_, in MOFs. The Spearman correlation coefficient, ρ, is given in both plots. The color bar shows the number of entries in each hexagonal bin.

On average, hypothetical MOFs in the QMOF database have larger cavity diameters and carbon fractions than synthesized MOFs (Figure S25), which, as we have shown, correlate with greater instability (Figure 5, Figure 7). However, these factors alone cannot explain the distinct distributions of Δ*E*_hull_ for hypothetical and synthesized MOFs in Figure 1B. If these factors were responsible, we would expect Δ*E*_form_ to show a similar distinction in Figure 1A, since both Δ*E*_form_ and Δ*E*_hull_ exhibit similar correlations with carbon fraction and cavity diameter. The indistinguishable Δ*E*_form_ distributions in Figure 1A suggest that Δ*E*_hull_ captures a fundamental difference in thermodynamic stability beyond carbon fraction or porosity, making it a more effective metric than Δ*E*_form_.

### Benchmarking Foundation Potentials with the QMOF-Thermo Database

The development of the QMOF-Thermo Database allows us, for the first time, to assess the accuracy of machine learning interatomic potentials (MLIPs) in predicting the thermodynamic stability of MOFs from a convex hull analysis. Here, we chose to investigate Meta’s Universal Model for Atoms (UMA)^74^ with the ODAC^75^ task (i.e., UMA-ODAC) and the equivariant Smooth Energy Network (eSEN)^76^ trained on ODAC25 (i.e., eSEN-ODAC25). UMA-ODAC was trained on a unified data set of materials, molecules, catalysts, organic molecular crystals, and MOFs via OMat24,^77^ OMol25,^78^ OMC25,^79^ OC20,^80^ and a subset of ODAC25,^75^ respectively, with ODAC chosen for the prediction task. In contrast, eSEN-ODAC25 was trained exclusively on ODAC25 and is a useful point of comparison for identifying the impact of a unified training set across material classes as implemented with UMA. To construct the convex hull phase diagrams, we used the pretrained MLIPs to relax each crystal structure (both the MOFs from the QMOF Database and the Materials Project structures within 0.01 eV/atom of the Materials Project-reported convex hull) and compared Δ*E*_hull_ from the resulting energies with those in the QMOF-Thermo Database.

From our thermodynamic analysis, we find that UMA-ODAC achieves a mean absolute error (MAE) of 0.059 eV/atom with respect to DFT for the predicted Δ*E*_hull_ values (Figure 8A), indicating that there is qualitative agreement but still room for improvement. For reference, the state-of-the-art models (e.g., eSEN-30M-OAM^76^) on Matbench Discovery^81^ currently predict the energy above hull of 0.016 eV/atom for inorganic bulk materials. With regards to predicting Δ*E*_form_ of MOFs, UMA-ODAC achieves an MAE of 0.077 eV/atom (Figure 8B). UMA-ODAC exhibits excellent agreement with respect to DFT in predicting the relaxed energies of MOFs in the QMOF Database on a per-atom basis, achieving an MAE of 0.006 eV/atom (Figure S26A). Even though the MOFs in ODAC25 differ from those in the QMOF Database, the model is able to generalize reasonably well across MOF chemical space.

The successes and shortcomings of UMA-ODAC in predicting MOF thermodynamic stability become clear when splitting up the analysis based on the model’s ability to describe MOFs derived from the QMOF database and materials along the convex hull derived from the Materials Project. As shown in Figure 8C, UMA-ODAC struggles to predict Δ*E*_form_ for the Materials Project-derived structures, resulting in an MAE of 0.548 eV/atom. This can be attributed to major deficiencies in the ability of UMA-ODAC in predicting the relaxed energy of the Materials Project-derived structures (Figure S26C, MAE = 0.468 eV/atom), which negatively impacts the Δ*E*_form_ calculations and the convex hull phase diagrams. The relatively poor performance in predicting the relaxed energies of Materials Project-derived structures with UMA-ODAC is perhaps not surprising, as there are no inorganic materials in ODAC25; nonetheless, the UMA training set includes OMat24, which consists of materials comparable to those in the Materials Project. As such, it is evident that the knowledge about material classes beyond the chosen prediction task (i.e., ODAC) is inherently limited.

To further evaluate the impact that the unified training set of UMA may have, we compared the thermodynamic stability predictions of UMA-ODAC (Figure 8) and those of eSEN-ODAC25 (Figure S27). On the basis of this analysis, we find that eSEN-ODAC25 predicts the energy above hull for the MOFs with an improved MAE of 0.025 eV/atom but does so via fortuitous error cancellation, as eSEN-ODAC25 struggles far more than UMA-ODAC in predicting the formation energies of both MOFs and Materials Project-derived materials (Figure S27). For both UMA-ODAC and eSEN-ODAC25, we trace much of the error in the formation energy predictions back to an extreme overstabilization of many of the elemental references (Figure S28), which makes the computed formation energies too endothermic on average.

**Figure 8:**
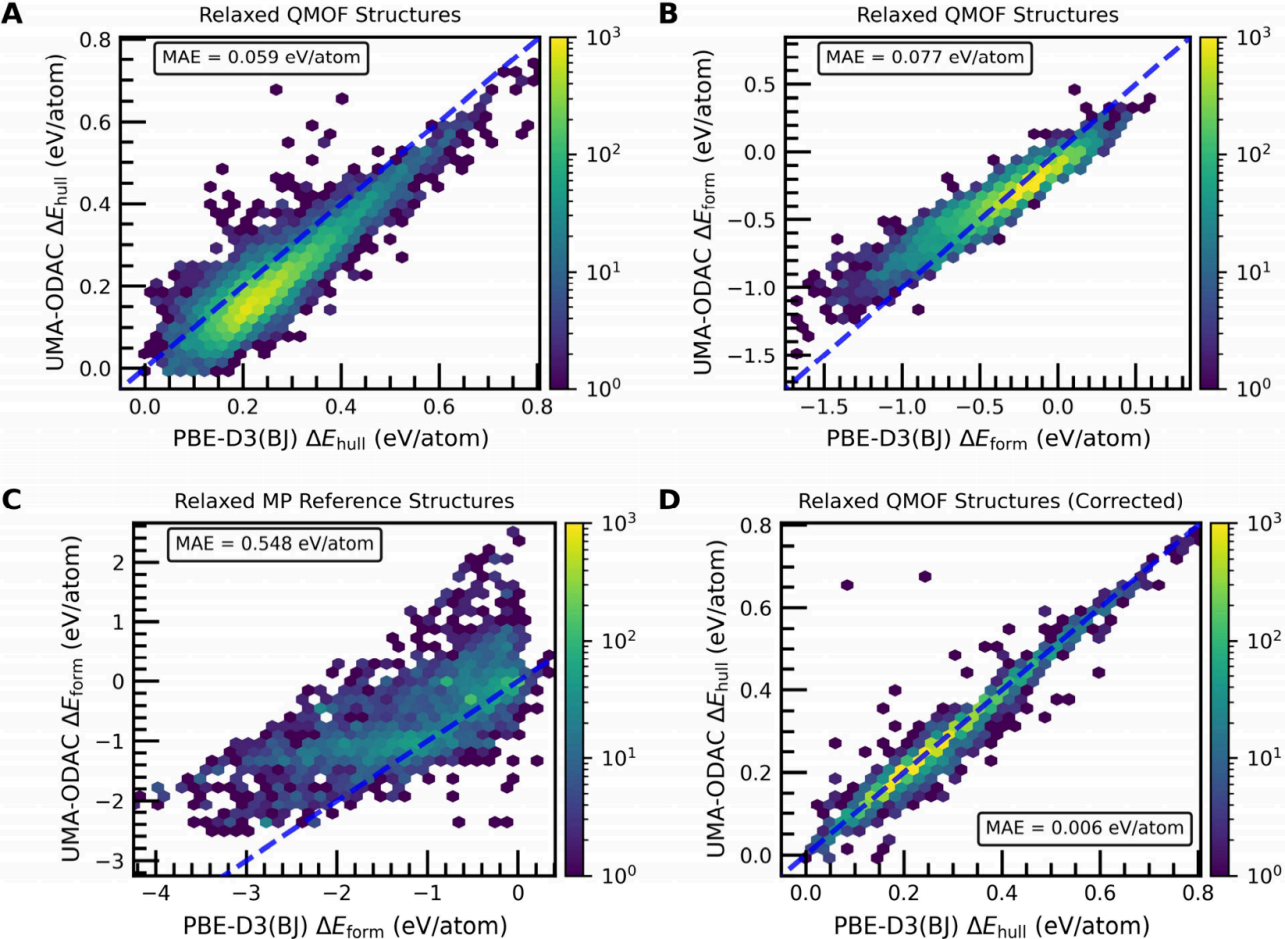
(A) Parity plot of Δ*E*_hull_ calculations between UMA-ODAC relaxations and DFT relaxations for the QMOF structures. (B) Parity plot of Δ*E*_form_ for QMOF and (C) Materials Project (MP) reference structures between UMA-ODAC relaxations and DFT relaxations. (D) Parity plot of Δ*E*_hull_ calculations using consistent DFT calculated reference structures between UMA-ODAC relaxations and DFT relaxations for QMOF structures. Mean absolute error (MAE) is calculated between the UMA-ODAC value and DFT value. The dashed blue line represents the line of parity. The color bar shows the number of entries in each hexagonal bin.

Based on these results, there are two main avenues to improve the performance of foundation potentials like UMA-ODAC or eSEN-ODAC25 in predicting MOF thermodynamic stability. The first route would be to train or fine-tune an MLIP on a single data set of both MOFs and their plausible decomposition products using an internally consistent level of theory. The second and more straightforward route, which does not involve any model retraining, would be to use DFT (at the same level of theory as the MLIP prediction task) to calculate the formation energy of the materials that reside on the convex hull. The DFT-derived energies of the materials that compose the convex hull can then be used as-is when computing MOF thermochemistry with the MLIP. Using the latter method reduces the MAE for the Δ*E*_hull_ and Δ*E*_form_ predictions of the MOFs to the error associated with the predictions of their relaxed energies: 0.006 eV/atom, which is an order of magnitude improvement (Figure 8D). The MOFs with large Δ*E*_hull_ errors predicted by UMA-ODAC can generally be traced back to radioactive elements that were excluded from the UMA model, in particular Pu. Finally, we used the corrected UMA-ODAC model to predict the thermodynamic stability of 895 randomly selected hypothetical MOFs in the ARC-MOF Database^48^ and show that the formation energy and energy above hull distributions remain largely unchanged compared to the hypothetical MOFs in the QMOF Database (Figure S30).

### Practical Considerations

Before concluding, we will reiterate several practical considerations when interpreting and applying the QMOF-Thermo Database in future studies. First and foremost, the data made available as part of the QMOF-Thermo Database solely describes thermodynamic factors governing MOF stability. As we have shown, virtually all MOFs are predicted to be thermodynamically metastable, such they can be considered kinetically trapped phases. Therefore, one should exercise caution in claiming that one MOF is more likely to be synthesized than another solely due to a lower Δ*E*_hull_, as kinetic factors may also play an important role. Absent other information, a more reliable use of Δ*E*_hull_ would be to exclude proposed structures that are thermodynamically infeasible. For a similar reason, GenAI models for MOFs should not optimize for a Δ*E*_hull_ near zero but rather a plausible distribution of Δ*E*_hull_ values like that shown in Figure 3. Furthermore, while the convex hull diagram can describe how unlikely a given MOF is to be realized, it generally does not directly relate to the MOF synthesis process itself, which is typically carried out solvothermally. Instead, the convex hull construction should only be interpreted as describing how (meta)­stable a real or proposed MOF is with respect to the set of thermodynamically stable decomposition products based on computed formation energies. In practice, these decomposition products are likely closer to what one might expect in a calcination process. Finally, the convex hull diagrams provided with the QMOF-Thermo Database do not currently account for finite temperature effects (with the exception of Figure S6) and are considered in a closed environment, both of which may influence the precise identity of the decomposition products.

## Conclusions

Through the use of high-throughput DFT calculations, we have computed the formation energy and convex hull phase diagrams for over 20,000 MOFs in the QMOF Database to describe their propensity toward phase transition and decomposition. On the basis of this analysis, we find that virtually no MOFs lie on the convex hull, indicating that MOFs are universally metastable materials. Although decomposition is a thermodynamically favorable process for virtually all MOFs, there necessarily exists an energetic barrier that makes them kinetically trapped in their metastable states. Throughout this work, we have also brought attention to the fact that a negative formation energy from the elements is not a suitable proxy for MOF thermodynamic stability and that the formation energy has little correlation with the energy above hull. We encourage the adoption of the energy above hull when predicting the thermodynamic stability of newly proposed MOFs and have demonstrated how the energy above hull can be used as a synthesizability metric for filtering computationally generated MOF structures.

Using the energy above hull as a stability metric, we identified numerous structural and chemical factors that contribute to the degree of metastability for MOFs. We confirmed that the thermodynamic penalty associated with porosity is inherent to all MOFs (synthesized or not) and grows until plateauing at sufficiently high porosities. Synthesized MOFs in the QMOF Database containing electron-rich, late transition metals are typically less thermodynamically stable than electron-poor, early transition metals. Many of the most thermodynamically stable MOFs contain hard metals bonded to hard carboxylate-containing ligands, which is a bond that is expected to be strong according to hard and soft acid base theory. Other thermodynamic stability trends related to metal identity and linker identity are present but are more confounded by each other. Nonetheless, we find that MOFs with a high carbon fraction have less favorable formation energies and energy above hull values due to elemental carbon being a strong thermodynamic sink, and a minimum exists for both thermodynamic properties as a function of increasing carbon fraction.

To democratize the results presented in this work, we have released the QMOF-Thermo Database,^43^ which includes the energy above hull calculations for the 20 000+ MOFs studied in this work and can be readily expanded upon with new DFT calculations. The QMOF-Thermo Database can also serve as a platform to benchmark machine learning interatomic potentials, which we have done for foundation potentials trained on the ODAC25 data set and found that it is critical for the training data to consist of both MOFs and the dense-phase inorganic structures that populate the hull. A simple *post hoc* correction of these foundation potentials by using the DFT-calculated energies of the inorganic materials composing the hull can make them applicable to MOFs without model retraining.

## Methods

### Density Functional Theory

All quantum-mechanical calculations were carried out using plane-wave, periodic DFT via the Vienna *Ab Initio* Simulation Package^82^ (VASP) using v.6.5.1 for all new calculations presented in this work. Unless otherwise stated, results are based on the exchange–correlation functional by Perdew, Burke, and Ernzerhof^83^ (PBE) with D3 dispersion corrections^84^ and Becke–Johnson (BJ) damping.^85^ For most of the MOFs considered in this work, the DFT-optimized structures and energies at the PBE-D3­(BJ) level of theory were available from the QMOF Database v16,^44,45^ which we adopted in this study. Unless otherwise stated, new calculations were generally carried out with QMOF-compatible settings. To briefly summarize, the QMOF-compatible settings include: a plane-wave kinetic energy cutoff at 520 eV, projector-augmented-wave^86^ (PAW) v.54 PBE pseudopotentials as described further in the original QMOF Database manuscript,^44,45^ a *k*-point grid density of 1000 per number of atoms, a maximum force tolerance of 0.03 eV/Å, an energy tolerance of 10^–6^ eV for converging the self-consistent field equations, and a high-spin magnetic initialization. Refer to the original QMOF Database publication for additional details.^44^

To ensure compatibility between calculations, the structures from the Materials Project (MP)^29^ needed to construct the convex hull were reoptimized using the PBE-D3­(BJ) level of theory (i.e., without + *U* corrections) and nearly identical settings as that used in the QMOF Database. The one notable difference was with regard to the *k*-point density, in which we took a more precise approach by adopting a bandgap-dependent *k*-point spacing to ensure the results for metallic systems were well-converged. The KSPACING parameter in VASP was adopted and was specified for each material via an empirical relationship^29,87^ based on the PBE/PBE+*U* bandgap provided by the Materials Project. The DFT-converged PBE/PBE+*U* atomic magnetic moments from the Materials Project were used as the initial guesses for calculations on the Materials Project structures in this work.

Motivated by prior work on the Materials Project,^28^ we also considered the use of the r^2^SCAN-D4 functional^88^ in constructing the convex hull diagrams. However, based on the recomputed convex hull diagrams for a random selection of Zn MOFs (including their corresponding Materials Project-derived reference structures), we found that the r^2^SCAN-D4 functional did not have a significant impact on the energy above hull for the MOFs (Figure S5). Since r^2^SCAN-D4 data are not present in the QMOF Database and would require a large recompute effort, we chose to adopt the PBE-D3­(BJ) level of theory in this work. Similarly, we also predicted the Gibbs free energy above hull for a random selection of Zn MOFs. This included the consideration of gaseous decomposition products and the use of machine learned interatomic potentials to apply thermal corrections to the DFT electronic energies, outlined in the Supporting Information methods. We found that the relative Gibbs free energy above hull values at room temperature and 1 bar pressure were not qualitatively different than the electronic energy above hull values, although notable deviations can be observed at elevated temperatures (Figure S6).

All newly carried out VASP calculations were performed in a high-throughput fashion via the Quantum Accelerator (QuAcc) 0.17.0 ^89^ that utilizes the Atomic Simulation Environment (ASE)^90^ v 3.25.0. Calculations were orchestrated using the Jobflow and Jobflow-Remote workflow tools.^91^ In the analysis of the DFT results, Zeo++ ^92,93^ v. 0.3 was used to calculate textural properties, and Pymatgen^94^ v. 2025.1.9 was used for convex hull construction and interpretation.

### QMOF Source Databases

The following is a description of the different data sources for the MOF structures found in the QMOF Database as a point of reference. MOFs in the QMOF Database have been classified as “synthesized” (i.e., based on experimentally reported crystal structures) or “hypothetical” (i.e., designed on a computer) based on their source database. Synthesized MOFs include those from the MOF subset of the Cambridge Structural Database^2^ (CSD); the Computation-Ready, Experimental (CoRE) MOF Database 2019,^95^ which was derived from pre-existing crystal structures; and select pyrene-containing MOFs that come from prior work by Kinik et al.^96,97^ Hypothetical MOFs include those from the BoydWoo^51^ data set, which used the topology-based crystal constructor ToBasCCo;^50^ the Genomic MOFs (GMOF) Database;^98^ trinuclear copper MOFs in the Topologically Based Crystal Constructor (ToBaCCo) MOF database;^53^ Zr and Hf MOFs derived from the Anderson et al.^54^ data set, which was constructed using ToBaCCo-3.0;^52^ the Mail-Order MOF-5 data set,^55^ which are hypothetical MOF-5 analogues using commercially available organic molecules; the hypothetical MOF-74 data set,^56^ which are hypothetical MOF-74 analogues using linkers found in PubChem. We note that the labeling adopted in this work does not imply that MOFs labeled as “hypothetical” are impossible to synthesize; rather, such structures are computationally constructed. A small fraction of the hypothetical MOFs may have coincidentally been synthesized. Our MOFid^49^ cross-reference with the CoRE MOF Database^3^ (2025 v1.0) identified six such MOFs. It is possible that more hypothetical MOFs than those identified have been synthesized; however, for simplicity we ignore such edge cases, as they are not expected to influence the main conclusions of this work.

### Convex Hull Phase Diagrams

To calculate the energy above hull, Δ*E*_hull_, for the 20,373 MOFs in v16 of the QMOF Database,^44,45^ it was necessary to compute the DFT total energies for the reference materials that make up the convex hull (i.e., the most thermodynamically stable materials, Δ*E*_hull_ = 0 eV/atom). These hull materials were drawn from each MOF’s chemical space (e.g., C–H–N–O–Zn): the collection of all known materials composed exclusively of one or more of the same elements (e.g., Zn, ZnO, CO_2_, H_2_O). For the MOFs in the QMOF Database, 1828 chemical spaces exist, which can be represented as 834 unique, top-level chemical spaces with no parents (e.g., C–H–N–O–Zn is a parent of C–H–O–Zn). Reference materials were all pulled from the Materials Project v2025.06.09, which includes the Δ*E*_hull_ for each material at varying levels of DFT theory. Since this report’s VASP settings differ from the Materials Project, reoptimizations were carried out for all Materials Project structures with a reported PBE/PBE+*U* Δ*E*_hull_ between 0 and 0.01 eV/atom to account for possible polymorph reordering and emergence/disappearance of convex hull structures. The upper bound of 0.01 eV/atom was chosen by incrementally increasing the upper bound and using DFT to calculate the change in Δ*E*_hull_ for all QMOF structures until the effect of including more structures was negligible (Figure S7). Consequently, for the 834 unique chemical spaces, the DFT-optimized structures and corresponding energies for a total of 7889 Materials Project reference materials needed to be calculated, which were subsequently used to find the Δ*E*_hull_ for the 20,373 MOFs in the QMOF Database. The convex hull phase diagrams throughout this study were calculated under the conditions of a closed system and in the solid state. Nonetheless, it is possible to build upon the data made available in the QMOF-Thermo Database to study open systems via a grand potential phase diagram^99,100^ or solid–aqueous equilibria via a Pourbaix diagram.^101,102^

### MLIP Benchmarking

To benchmark state-of-the-art MLIPs in predicting the Δ*E*_hull_ of MOFs, we constructed convex hull diagrams using relaxed structures and energies determined by Meta’s UMA-ODAC^74^ (uma-s-1p1) and eSEN-ODAC25 ^75,76^ (esen_sm_odac25_full) MLIPs. To ensure the total energies are comparable between the DFT calculations and MLIPs, MOFs with elements that contain mismatched pseudopotentials between ODAC25 and the QMOF Database were excluded, as described in the Supporting Information, resulting in 15,873 MOFs for analysis. For these MOFs, there exist 885 chemical spaces, which can be represented as 377 unique, top-level chemical spaces. Mirroring the DFT convex hull construction, 3550 reference materials with a reported Δ*E*_hull_ between 0 and 0.01 eV/atom were obtained directly from the PBE/PBE+*U* data on the Materials Project. Using UMA-ODAC and eSEN-ODAC25, MLIP relaxations of both the MOFs and reference materials were carried out until a maximum force threshold of 0.03 eV/Å was reached. The atomic positions, cell shape, and cell volume were allowed to vary simultaneously during the relaxation. The Broyden–Fletcher–Goldfarb–Shanno (BFGS) algorithm was used as the optimizer, and ASE’s FrechetCellFilter was used to enable cell relaxations. The MLIP-calculated energies of the local minimum energy structures were used to construct the convex hull and thereby determine the Δ*E*_hull_ values, which were compared to the analogous DFT values.

## Supplementary Material



## Data Availability

All data needed to reproduce and expand upon the convex hull phase diagrams are made available via the QMOF-Thermo Database on Figshare (https://doi.org/10.6084/m9.figshare.13147324). Additional Python scripts associated with this work can be found on GitHub at https://github.com/Quantum-Accelerators/qmof_thermo and have a mirror on Zenodo at https://doi.org/10.5281/zenodo.18869579. The VASP files can be found on NOMAD^103^ at https://doi.org/10.17172/NOMAD/2026.03.16-1.

## Notes

Supplementary methods, pedagogical diagrams, Gibbs free energy above hull results, r^2^SCAN-D4 comparisons, and additional figures, tables, analysis to complement the main text (PDF)
