# Supplementary material for: Predicting the Thermodynamic Limits of Metal–Organic Framework Metastability
Source: J Am Chem Soc. 2026 May 11;148(19):19487–501. doi: 10.1021/jacs.5c20253 (PMC13195659; doi:10.1021/jacs.5c20253)
Supplement: Supplementary file 1 [file ja5c20253_si_001.pdf]

Supporting Information

Predicting the Thermodynamic Limits of Metal–Organic Framework Metastability

Blake Dallmann,[1] Aryan Saha,[2] Andrew S. Rosen[1,*]

[1]Department of Chemical and Biological Engineering, Princeton University, Princeton, 08544, USA
[2]Department of Electrical and Computer Engineering, Princeton University, Princeton, 08544, USA
[*]asrosen@princeton.edu

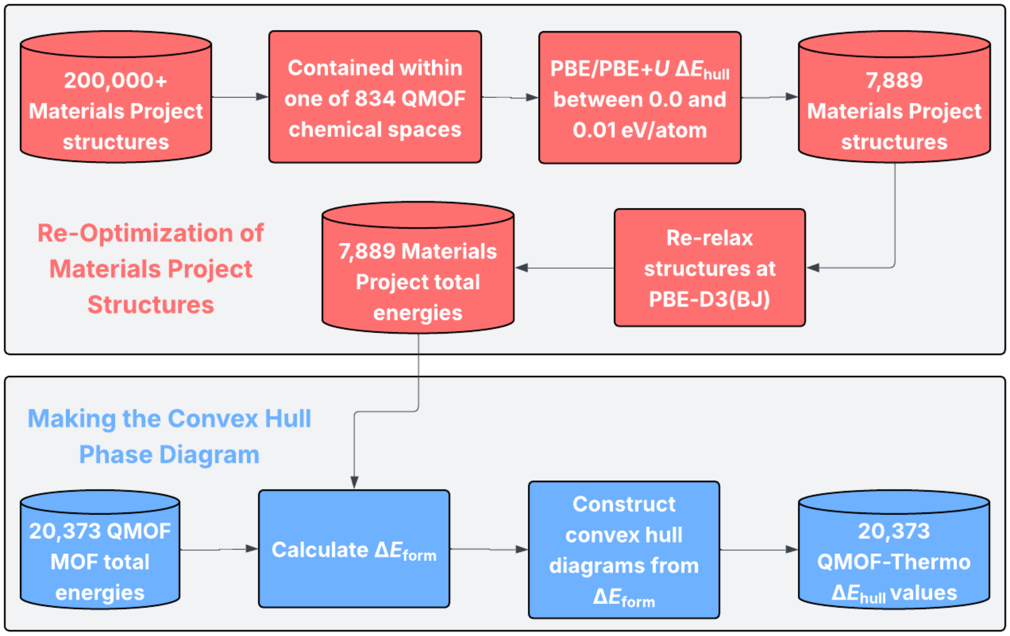

Figure S1. Workflow for re-relaxing structures from the Materials Project and constructing the convex hull diagrams in building the QMOF-Thermo Database.

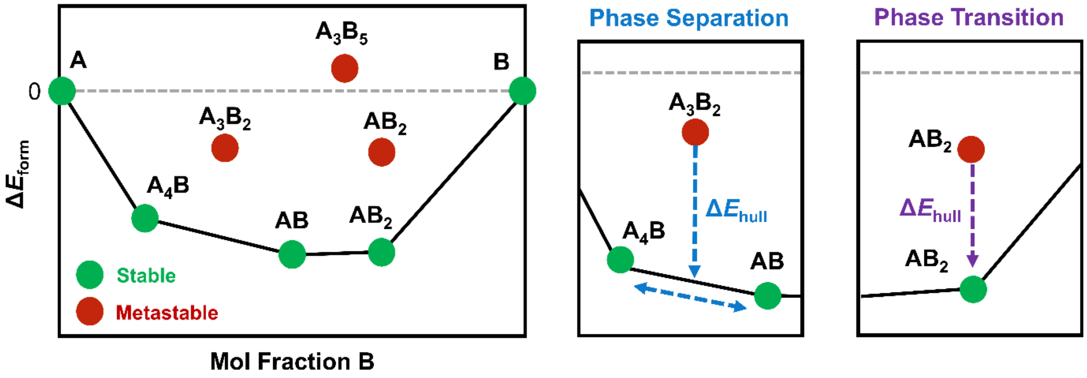

Figure S2. Example convex hull phase diagram for the hypothetical chemical space A–B. ΔE_{form} represents the formation energy from the elements for each material. The decomposition and phase transition reactions proceed through $3A_3B_2 \rightarrow 5AB + A_4B$ and $AB_2 \rightarrow AB_2$, respectively.

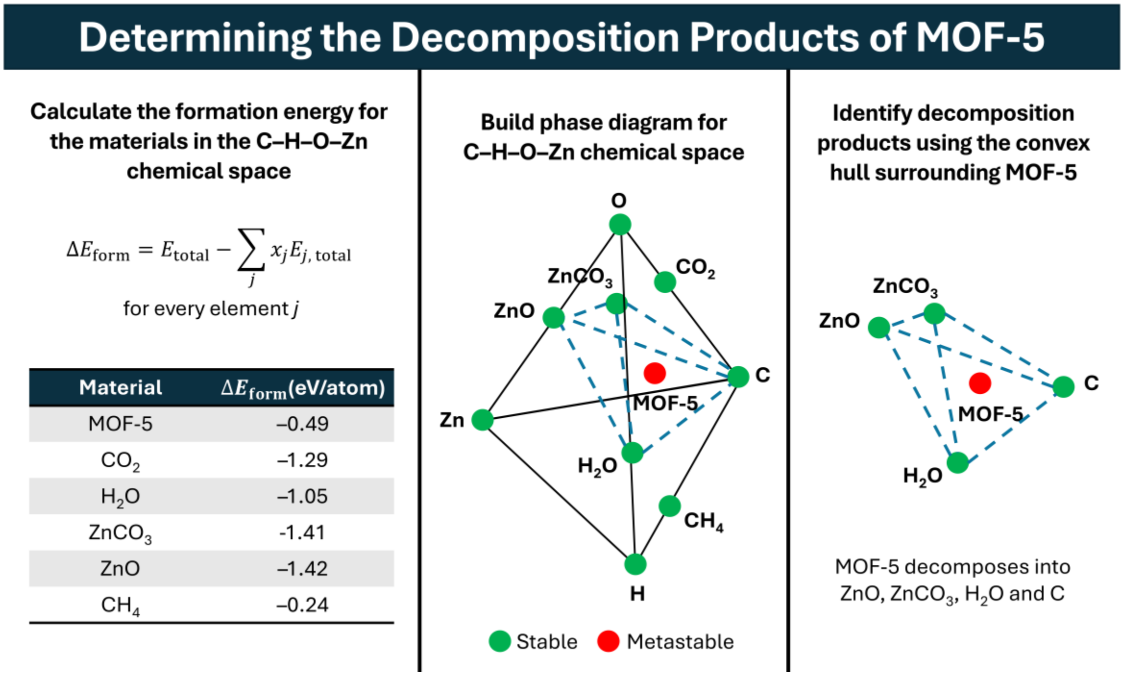

Figure S3. Schematic for determining the decomposition products of MOF-5.

$$\sum_i w_i = 1 \tag{1}$$

$$\sum_i w_i x_{i,\,j} = x_{\mathrm{MOF},\,j} \tag{2}$$

$$\Delta E_{\mathrm{MOF,\,hull}} = \Delta E_{\mathrm{MOF,\,form}} - \sum_i w_i \Delta E_{i,\,\mathrm{form}} \tag{3}$$

Equation S1. System of equations for calculating energy above hull (ΔE_{hull}) of a MOF. Here, w_i is the weight of decomposition product i, which represents the proportion of the MOF that decomposes into product i. These decomposition products are identified using the convex hull surrounding the MOF in the phase diagram (Figure S3). $x_{i,\,j}$ represents the elemental fraction of element j in decomposition product i. w_i and $x_{i,\,j}$ are determined by solving (1) and (2) as a system of equations. Once the w_i are obtained, $\Delta E_{\mathrm{MOF,hull}}$ is determined in equation (3) using w_i and $\Delta E_{i,\,\mathrm{form}}$, which is the formation energy of decomposition product i.

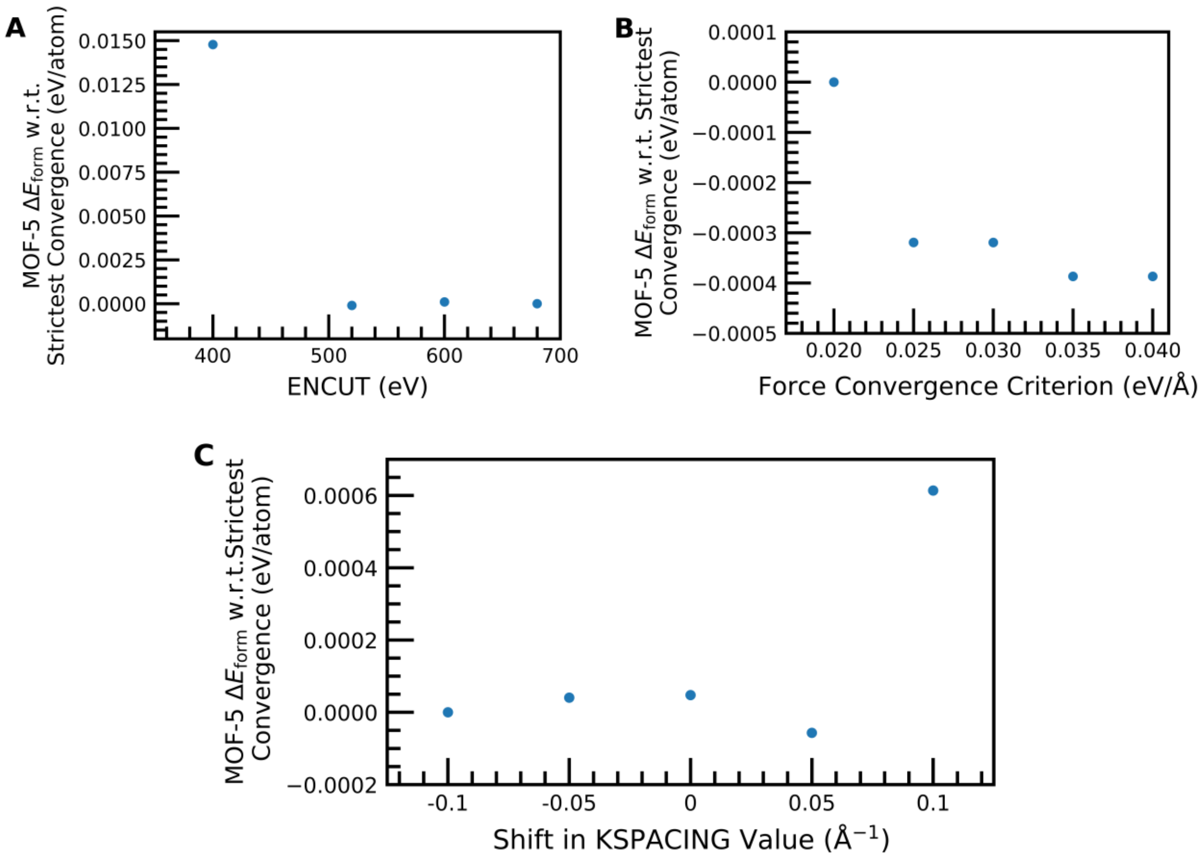

Figure S4. The ΔE_{form} for MOF-5 plotted against the VASP (A) ENCUT, (B) force convergence, and (C) KSPACING parameter. Shift in KSPACING Value = 0 represents the material-dependent KSPACING value that is determined empirically using the bandgap of the material. To calculate the ΔE_{form} of MOF-5, we predicted the total energy of the elemental ground state structures from the Materials Project (H_2, O_2, Zn, and C) and MOF-5. Apart from the specific parameter given in the plot, these DFT calculations used the parameters outlined in Methods–Density Functional Theory.

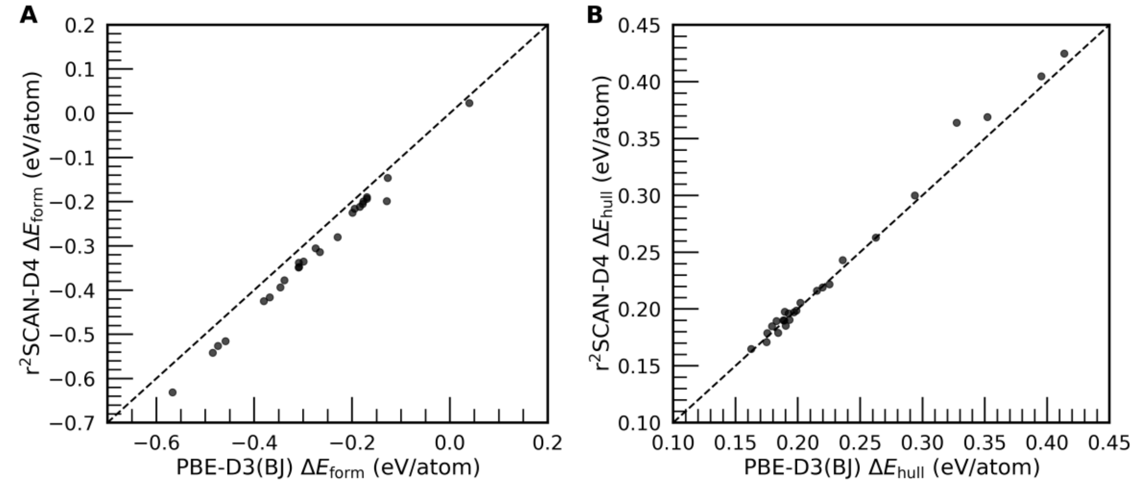

Figure S5. Parity plots of the PBE-D3(BJ) and r^{2}SCAN-D4 functionals for calculations of (A) formation energy from the elements (ΔE_{form}) and (B) energy above hull (ΔE_{hull}) for 25 random C–H–N–O–Zn MOFs.

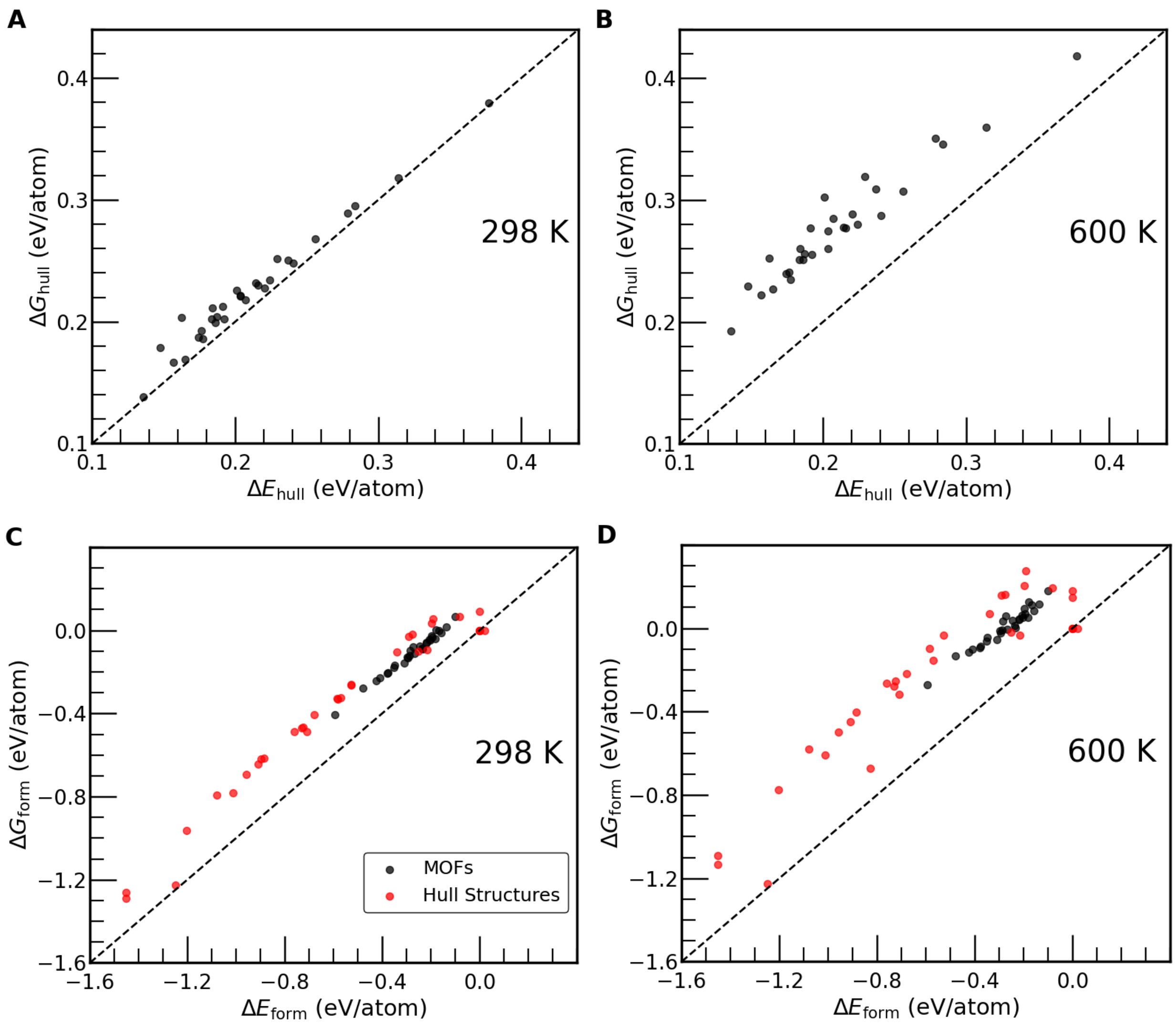

Figure S6. (A, B) Parity plots of the energy above hull for 30 random C–H–N–O–Zn MOFs using electronic DFT energy (ΔE_{hull}) and Gibbs free energy (ΔG_{hull}) at (A) 298 K and (B) 600 K. (C, D) Parity plots of the formation energy for 30 C–H–N–O–Zn MOFs and select hull structures in the C–H–N–O–Zn chemical space using ΔE_{form} and ΔG_{form} at (C) 298 K and (D) 600 K. Hull structures consist of crystalline Materials Project structures or gases that were on the hull for either the electronic ($\Delta E_{hull} = 0$ eV/atom) or Gibbs free energy ($\Delta G_{hull} = 0$ eV/atom) convex hull diagrams. All reported Gibbs free energies are at the given temperature and 1 bar pressure.

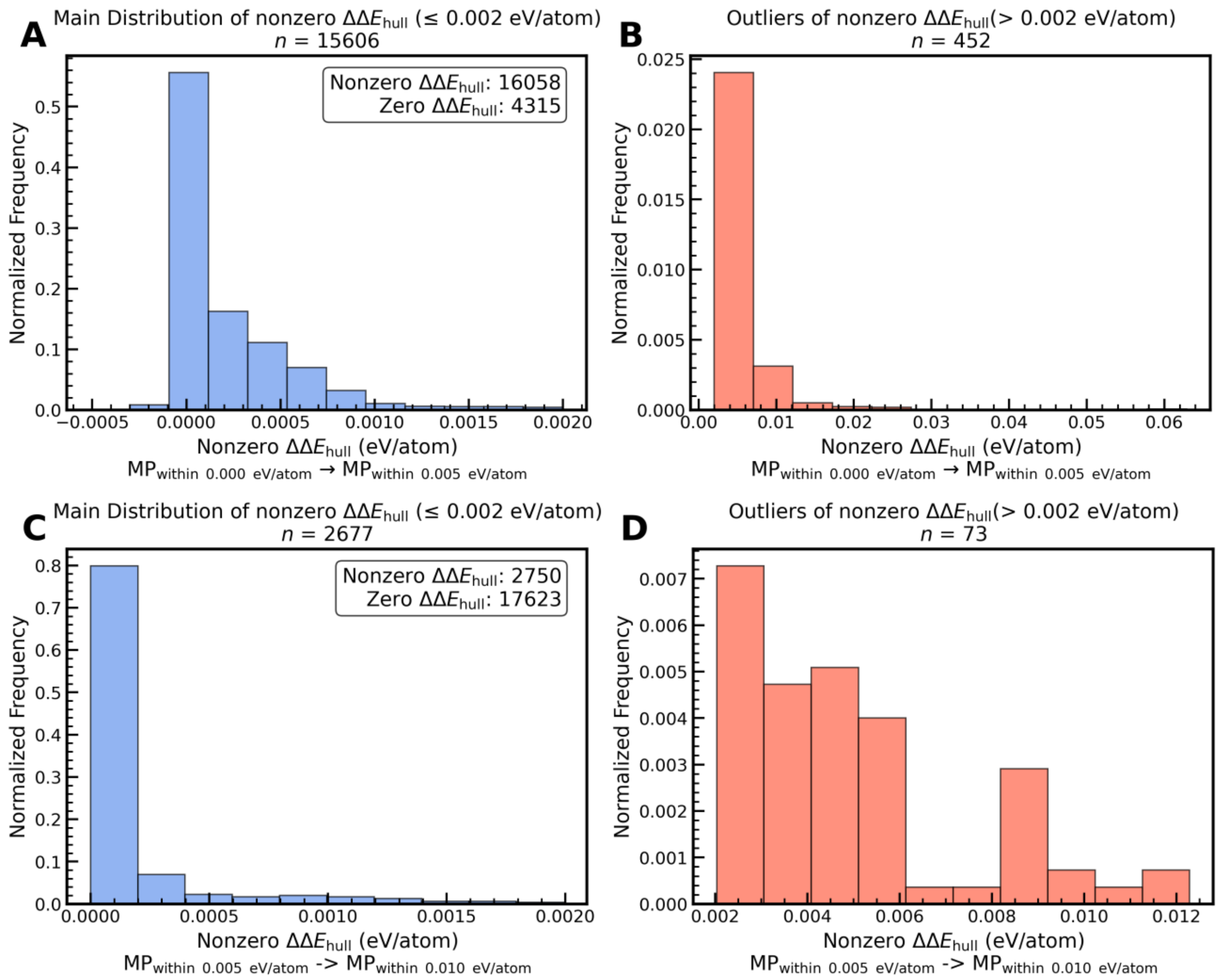

Figure S7. (A) Histogram of the change in ΔE_{hull} ($\Delta\Delta E_{\mathrm{hull}}$) when the convex hull basis is expanded from MP structures with $\Delta E_{\mathrm{hull}} = 0$ eV/atom (5047) to those with $\Delta E_{\mathrm{hull}} \leq 0.005$ eV/atom (6781). Outliers are given in (B). (C) Histogram of the change in ΔE_{hull} ($\Delta\Delta E_{\mathrm{hull}}$) when the convex hull basis is expanded from MP structures with $\Delta E_{\mathrm{hull}} \leq 0.005$ eV/atom (6781) to those with $\Delta E_{\mathrm{hull}} \leq 0.010$ eV/atom (7889).

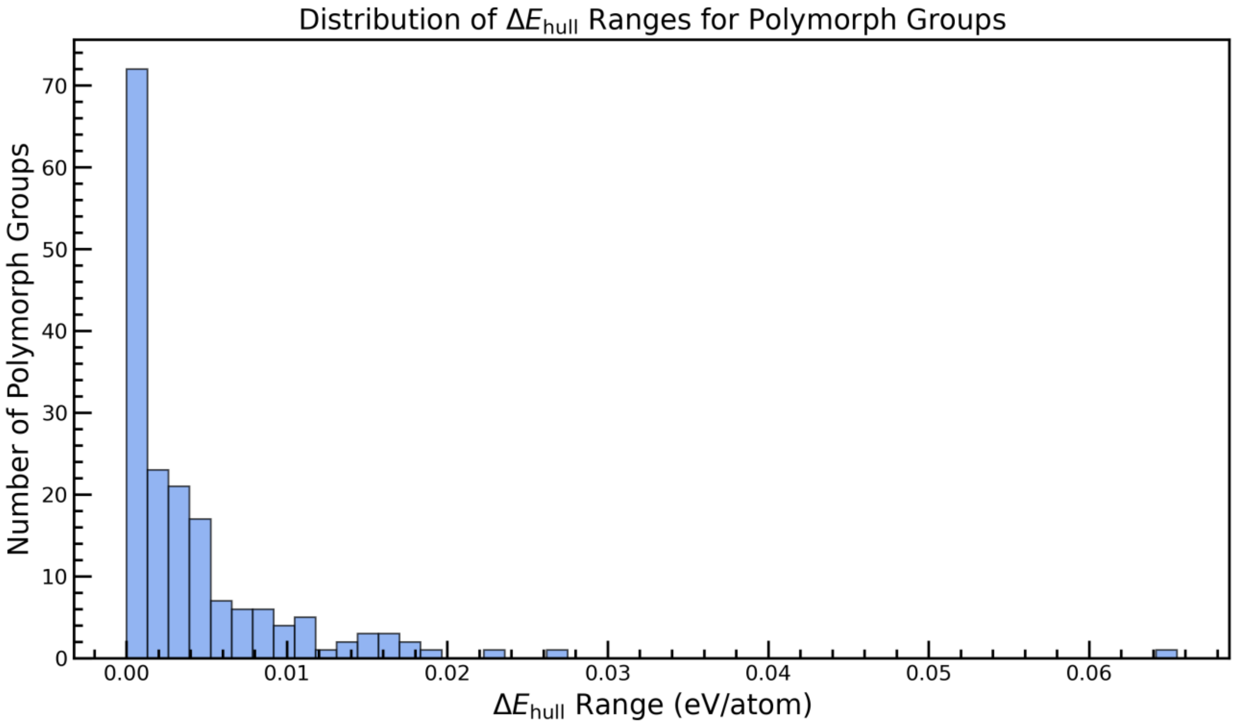

Figure S8. Distribution of the range in ΔE_{hull} values for the 176 polymorph sets in the QMOF database.

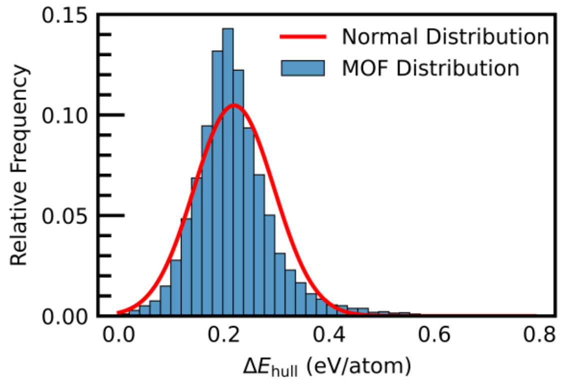

Figure S9. Histogram of ΔE_{hull} values for synthesized MOFs in the QMOF Database (16,882) compared to a normal distribution. The red line represents the height a bar would have at a given value of ΔE_{hull} if the distribution of ΔE_{hull} values was a perfect normal distribution.

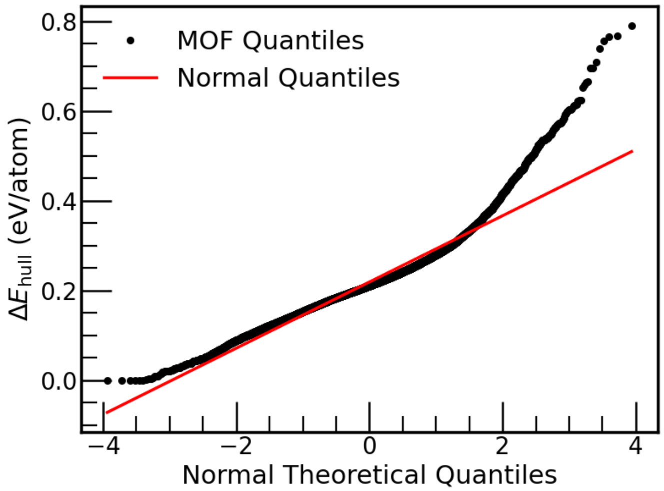

Figure S10. Q–Q plot of the ΔE_{hull} for synthesized MOFs in the QMOF Database (16,882). Black points show the empirical quantiles of MOF energy above hulls plotted against the theoretical quantiles of a normal distribution. The red line indicates perfect normal agreement.

Table S1. The value of ΔE_{hull} at different empirical quantiles in the synthesized MOF distribution

Quantile in Synthesized MOFs	0.80	0.90	0.95	0.99
ΔE_{hull} (eV/atom)	0.267	0.306	0.353	0.476

The value of ΔE_{hull} at different empirical quantiles in the population of synthesized MOFs in the QMOF Database.

Table S2. Hypothetical MOFs from QMOF that have been coincidentally synthesized

QMOF id	Name	ΔE_{hull}	DOI
qmof-53c7ebf	Zr-UiO-66	0.185	https://doi.org/10.1039/C5CP03920G
qmof-be54856	NbOFFIVE-2-Cu-i (ZU-62)	0.334	http://dx.doi.org/10.1002/anie.201913245
qmof-c07a718	New 1-ZnSiF6	0.299	http://dx.doi.org/10.1039/b815695f
qmof-c0b261d	UiO-67	0.206	http://dx.doi.org/10.1039/C4DT02582B
qmof-c406647	IRMOF-3	0.198	http://dx.doi.org/10.5012/bkcs.2010.31.04.1041
qmof-fb69c56	IRMOF-18	0.161	https://doi.org/10.1021/ja049408c

MOFs listed as hypothetical in the QMOF database that have been coincidentally synthesized. Structures were identified by comparing all hypothetical MOFids[1] listed in QMOF with those listed in the CoRE MOF Database[2] (2025 v1.0).

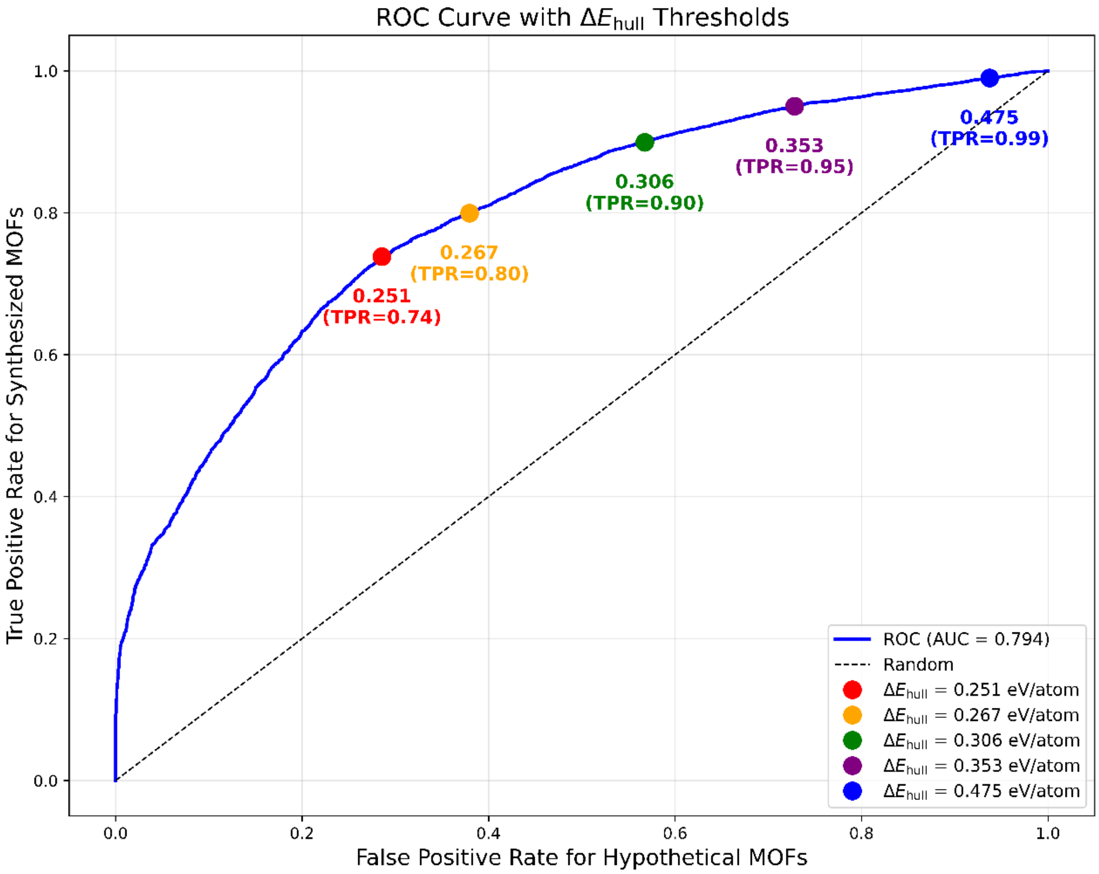

Figure S11. Receiving operative characteristic (ROC) curve for using ΔE_{hull} as a synthesizability threshold. The true positive rate (TPR) for synthesized MOFs is plotted against the false positive rate (FPR) for hypothetical MOFs in the QMOF database. The difference TPR – FPR reaches a maximum of 0.45 when a ΔE_{hull} threshold of 0.251 eV/atom is used. The area under the ROC curve (AUC) is 0.794. This analysis assumes hypothetical MOFs are negative datapoints. However, hypothetical MOFs in the QMOF database cannot be labeled as true negative datapoints, as some MOFs labeled as hypothetical may have coincidentally been synthesized or could eventually be synthesized.

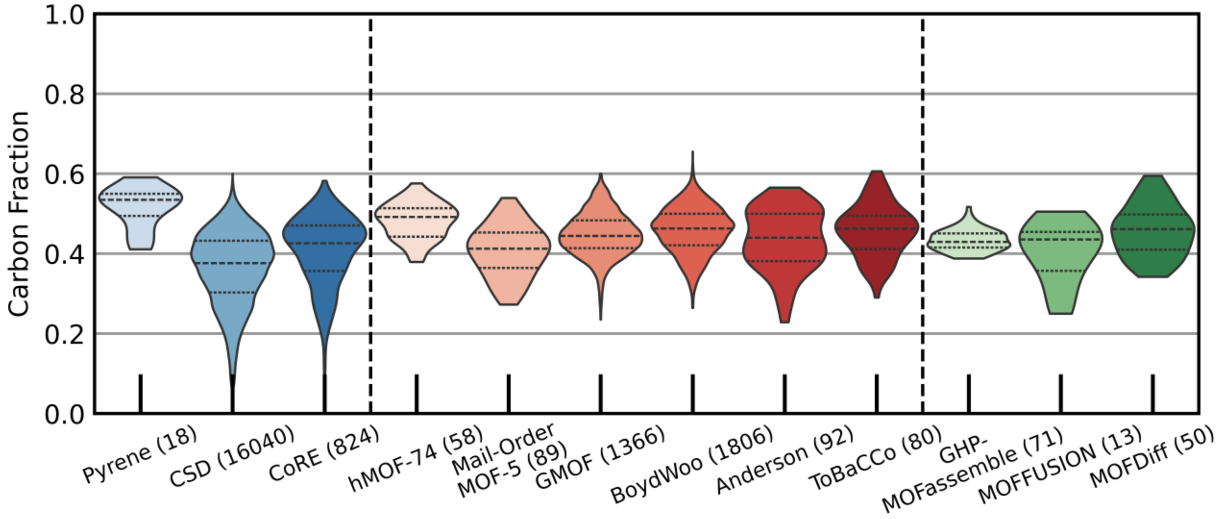

Figure S12. Violin plots of the carbon fraction for MOFs in the QMOF Database sorted by source database and AI-generated MOFs sorted by model. The total number of MOFs corresponding to a source or model is given in parentheses. MOFs from synthesized databases are in shades of blue (left), MOFs from hypothetical databases are in shades of red (middle), and MOFs from generative AI models are in shades of green (right). The central dashed line in each violin represents the median, and the dotted lines represent the first quartile and third quartile in each distribution.

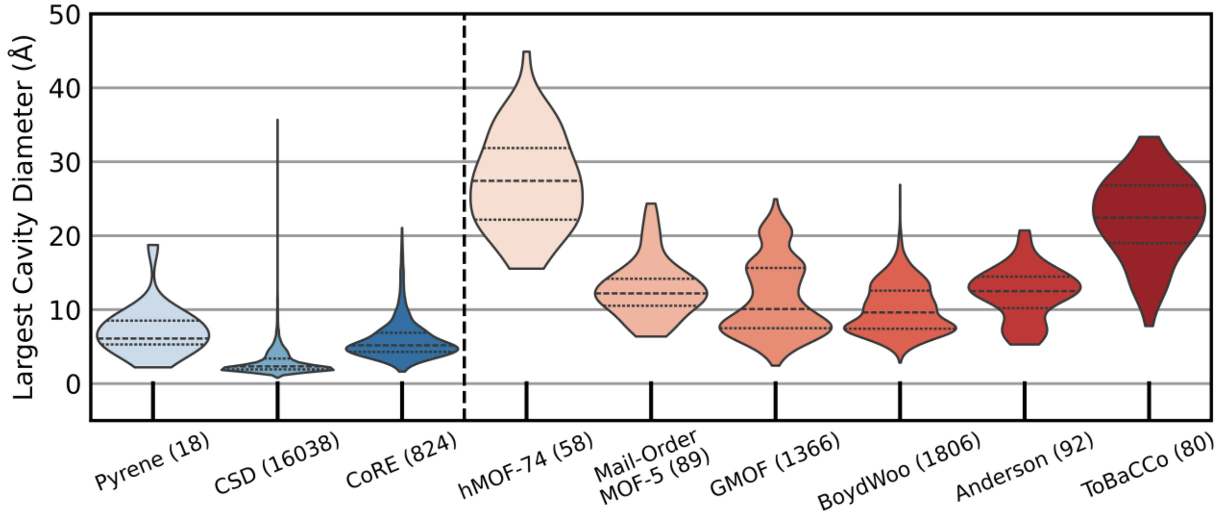

Figure S13. Violin plots of the largest cavity diameter for MOFs in the QMOF Database sorted by source database. The total number of MOFs corresponding to a source or model is given in parentheses. MOFs from synthesized databases are in shades of blue (left), MOFs from hypothetical databases are in shades of red (right). The central dashed line in each violin represents the median, and the dotted lines represent the first quartile and third quartile in each distribution.

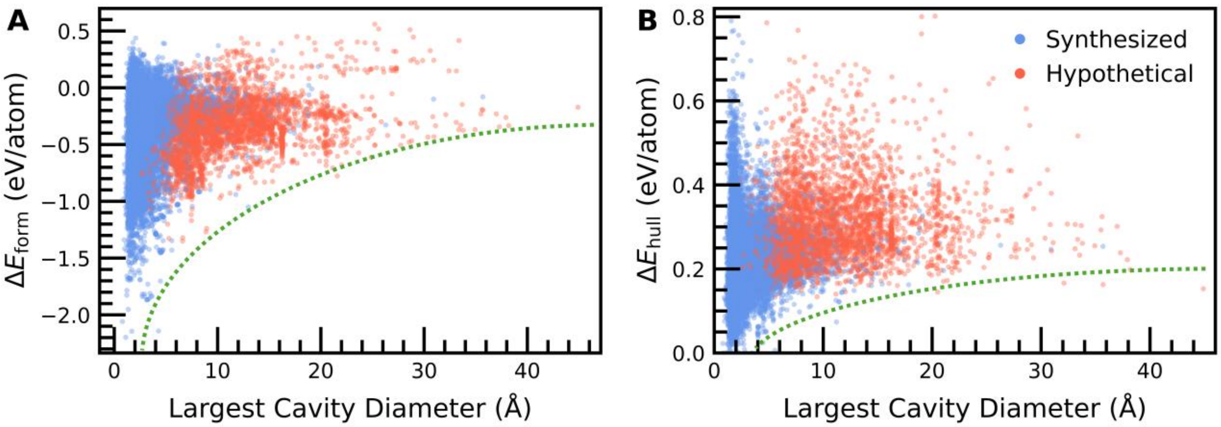

Figure S14. (A) ΔE_{form} and (B) ΔE_{hull} as a function of largest cavity diameter for synthesized and hypothetical MOFs in the QMOF Database. The dotted green lines are a guide for the eye.

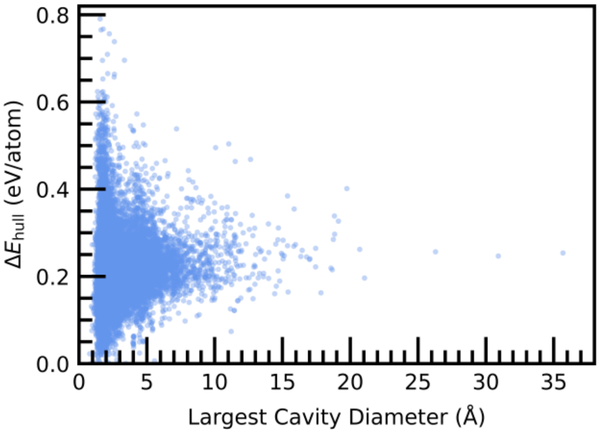

Figure S15. ΔE_{hull} and as a function of largest cavity diameter for only synthesized MOFs in the QMOF Database.

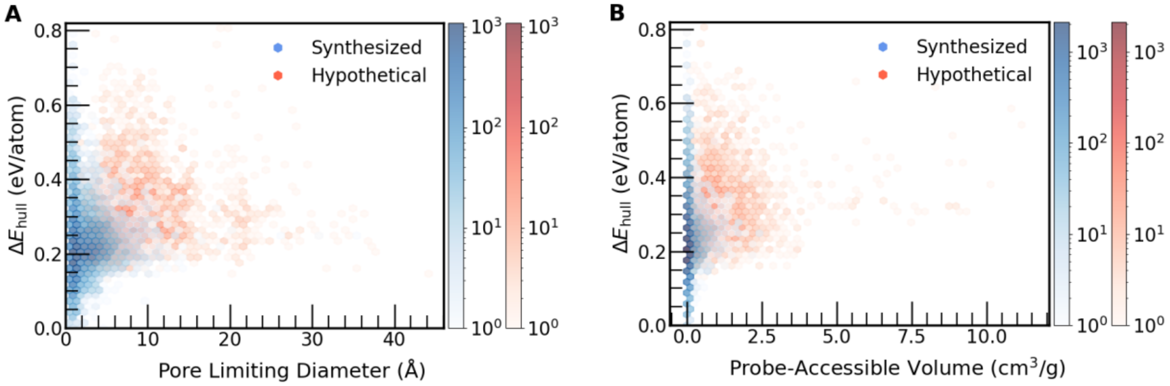

Figure S16. ΔE_{hull} as a function of (A) pore-limiting diameter (PLD) and (B) probe-accessible volume (PAV). Pore-limiting diameter is defined as the diameter of the largest sphere that can freely diffuse across the material. Probe-accessible volume is defined as the material's volume accessible to the entire volume of a spherical N_2 molecule with a radius of 1.86 Å. Both PLD and PAV were calculated using Zeo++. Data is for all MOFs in the QMOF Database.

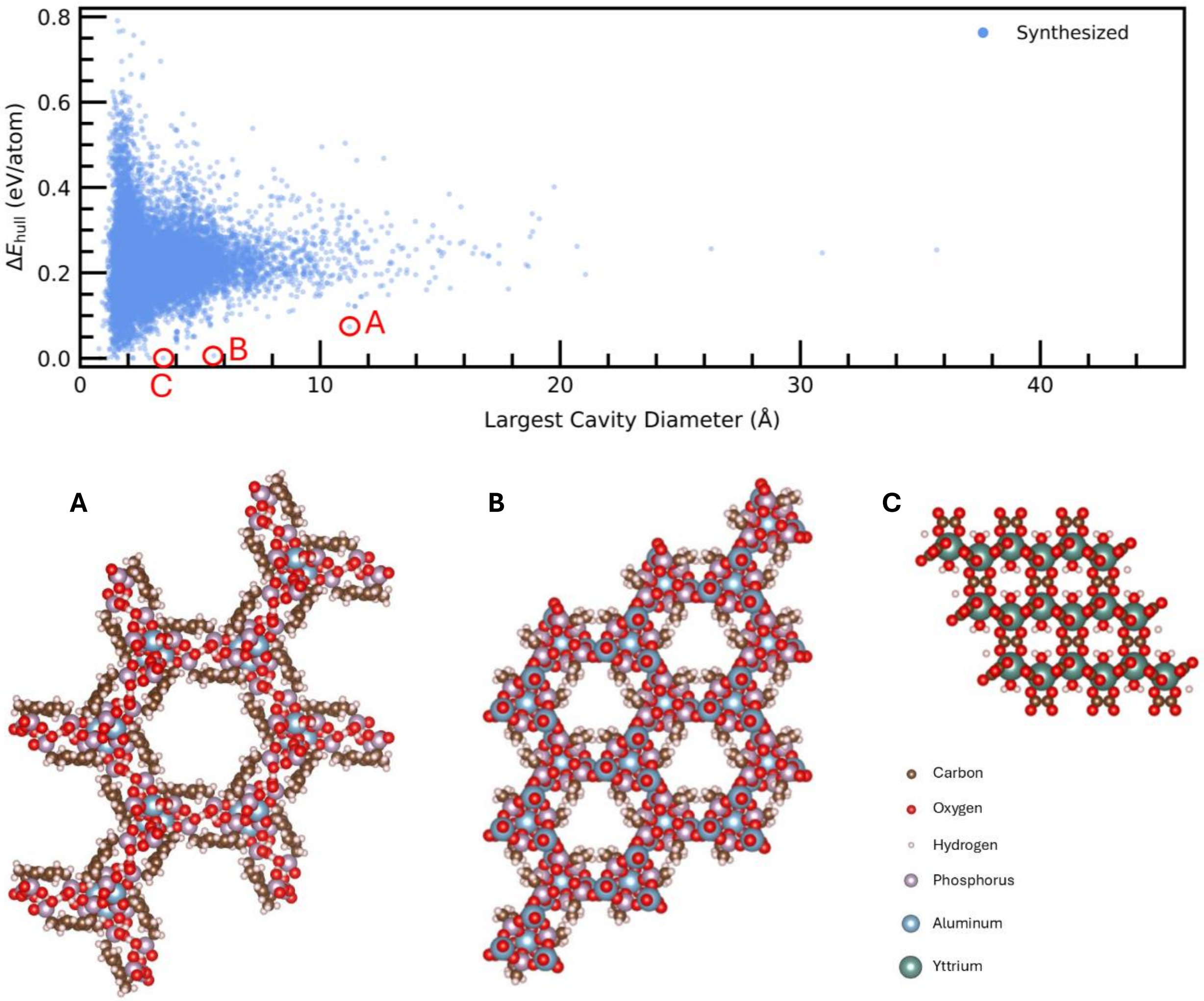

Figure S17. Outliers present in Figure 5B and Figure S15: (A) qmof-0205f07 at ΔE_{hull} = 0.074 eV/atom and LCD = 11.2 Å, chemical formula is $AlP_3H_{20}(C_6O_5)_2$, an aluminophosphate, (B) qmof-723a15f at ΔE_{hull} = 0.006 eV/atom and LCD = 5.6 Å, chemical formula is $Al_2P_3H_9(CO_3)_3$, an aluminophosphate, and (C) qmof-ff37c01 at ΔE_{hull} = 0.0004 eV/atom and LCD = 3.5 Å, chemical formula is $[Y(H_2O)]_2(C_2O_4)(CO_3)_2$, only has oxalate linkers.

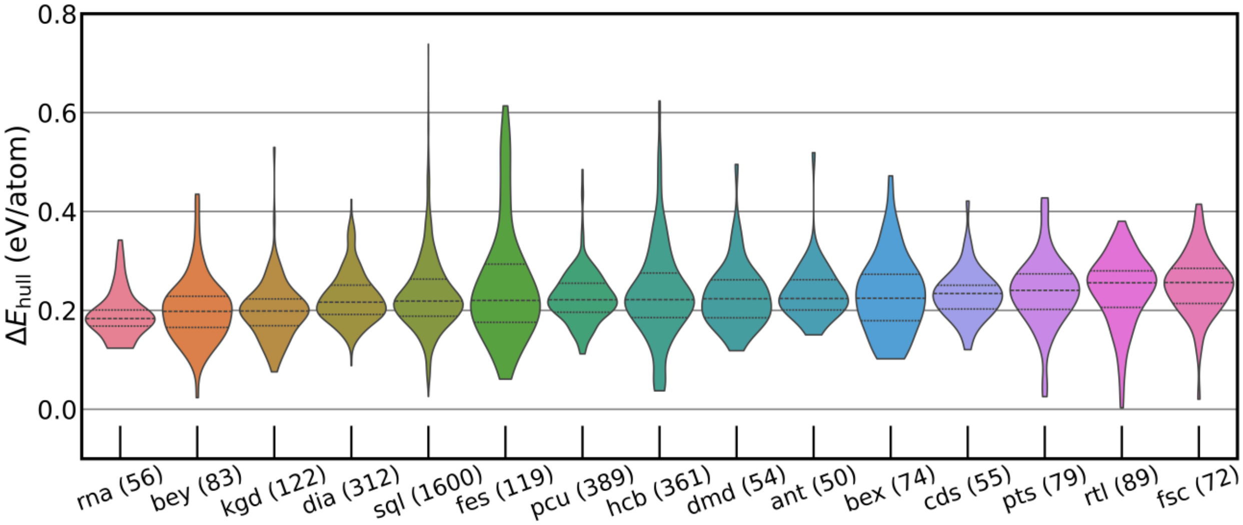

Figure S18. Violin plots of ΔE_{hull} for synthesized MOFs in the QMOF Database as a function of topology. Only topologies with 50 or more MOFs in the QMOF Database are shown.

Table S3. The median ΔE_{hull} of synthesized MOFs in the QMOF Database sorted by topology as determined from MOFid.[1] Only topologies with 10 or more MOFs in the QMOF Database are shown.

**Topology** **(# MOFs)**	ΔE_{hull} **(eV/atom)**				
		**mog** (12)	0.207	**bex** (74)	0.225
		**hex** (15)	0.210	**rob** (28)	0.225
**raa** (11)	0.140	**hxl** (25)	0.214	**bnn** (15)	0.226
**dmc** (12)	0.164	**dia** (312)	0.217	**ins** (12)	0.227
**rna** (56)	0.183	**sod** (26)	0.217	**tfz-d** (14)	0.231
**sra** (42)	0.185	**nbo** (48)	0.218	**cpr** (11)	0.232
**qtz** (14)	0.192	**sql** (1600)	0.219	**kgm** (15)	0.233
**crb** (15)	0.193	**fes** (119)	0.220	**mab** (21)	0.234
**bey** (83)	0.198	**pcu** (389)	0.222	**hms** (14)	0.234
**kgd** (122)	0.199	**neb** (10)	0.222	**cds** (55)	0.234
**tfi** (18)	0.199	**ths** (31)	0.222	**pts** (79)	0.240
**bbf** (18)	0.200	**lvt** (29)	0.222	**rtl** (89)	0.256
**srs** (20)	0.202	**hcb** (361)	0.222	**fsc** (72)	0.256
**met** (13)	0.203	**dmd** (54)	0.224	**apo** (14)	0.260
**dmp** (14)	0.205	**ant** (50)	0.224	**bcu** (29)	0.260

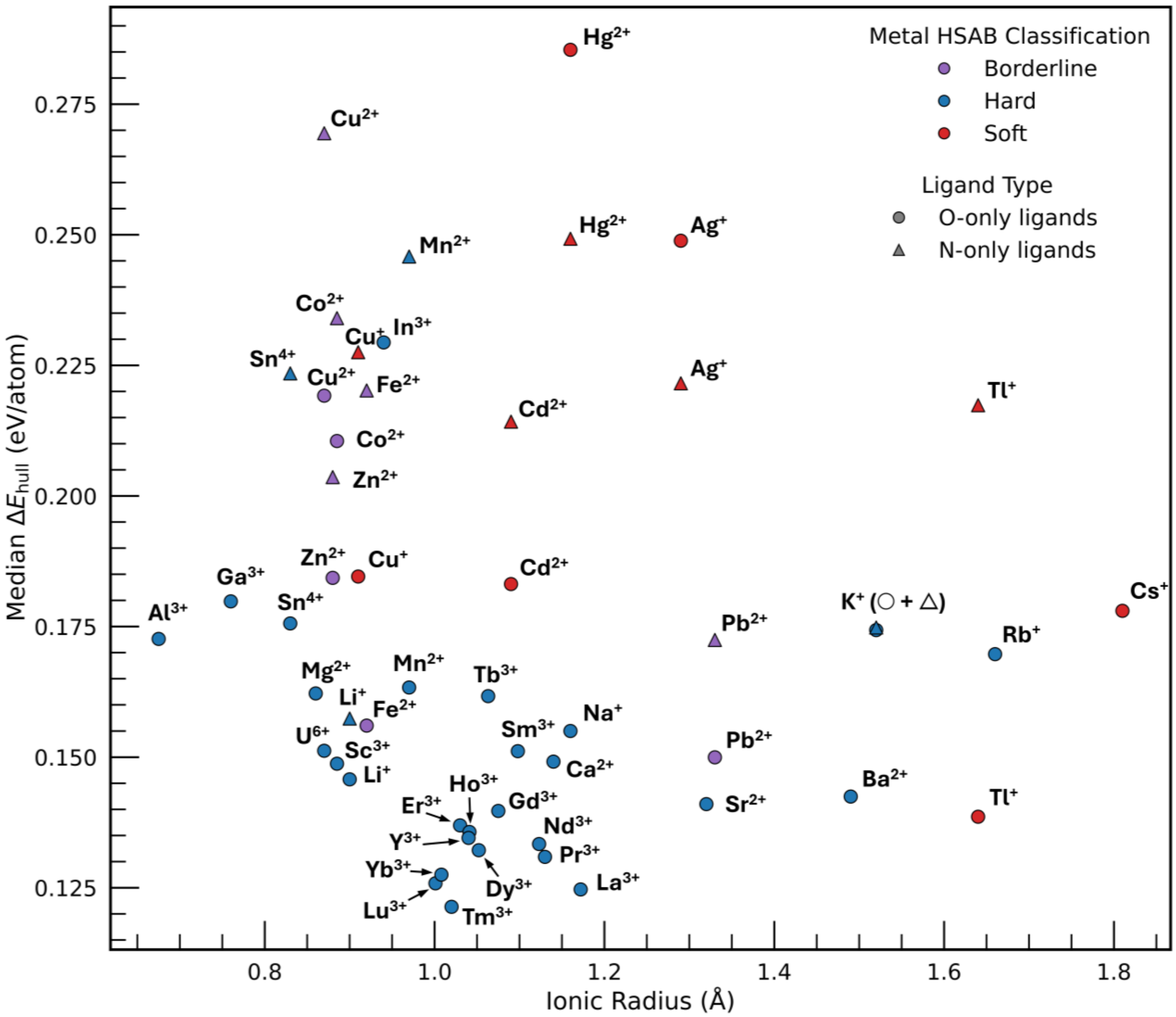

Figure S19. Median ΔE_{hull} as a function of ionic radius for synthesized MOFs in the QMOF Database organized by metal cation, linker type, and HSAB classification. For the O-only and N-only linker types, only M–C–H–O and M–C–H–N chemical systems were included, respectively, to ensure the Lewis base of interest was directly binding to the metal Lewis acid in the MOF. Structures with multiple metal cation identities or with less than eight entries are excluded. See Supplementary Methods for details on how oxidation state and HSAB were assigned.

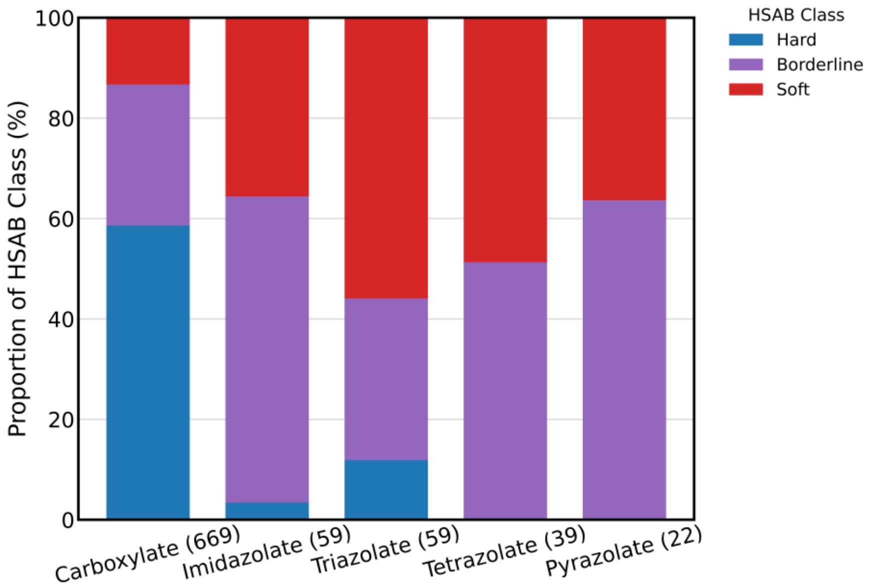

Figure S20. Proportion of hard/soft/borderline metal ions in the linker-based ΔE_{hull} distributions given in Figure 6C. The number in paratheses is the number of MOFs in that distribution. Only monometallic MOFs with a metal oxidation state that can be identified by oxiMACHINE are shown, which is why the number of entries for each linker type is less than the entries present in Figure 6C.

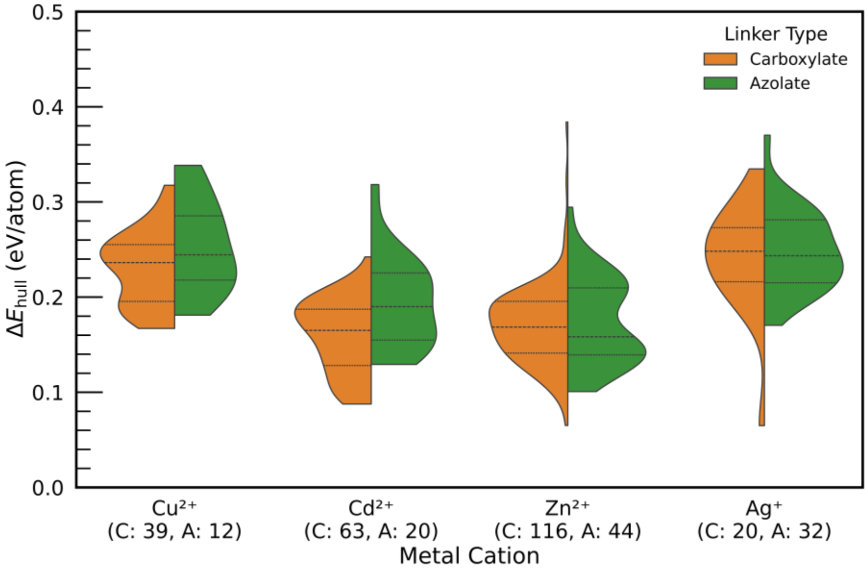

Figure S21. ΔE_{hull} distributions of synthesized MOFs containing late transition metals and only carboxylate or azolate linkers in the QMOF Database. "Azolate" describes any MOF that has only pyrazolate, imidazolate, triazolate, or tetrazolate-containing linkers. MOFs with multiple azolate linker types or different azolate functional groups on a single linker are included under the category of "azolate".

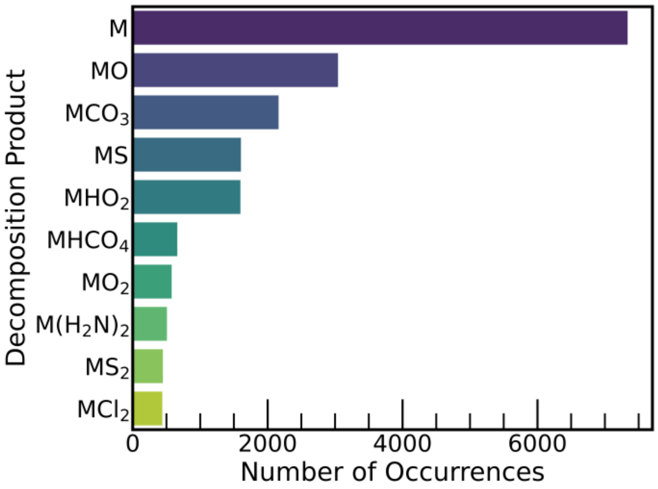

Figure S22. Number of occurrences for the ten most frequent metal-containing MOF decomposition products (M = metal atom) in the QMOF-Thermo Database.

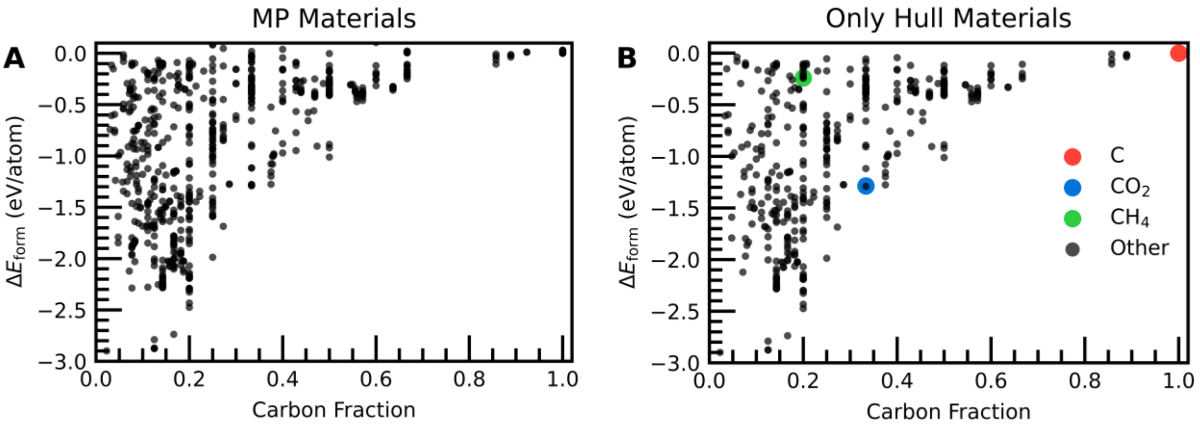

Figure S23. ΔE_{form} as a function of stoichiometric carbon fraction for (A) all re-optimized carbon-containing Materials Project structures (668 materials) and (B) only the materials that lie on the convex hull (ΔE_{hull} = 0 eV/atom) (402 materials).

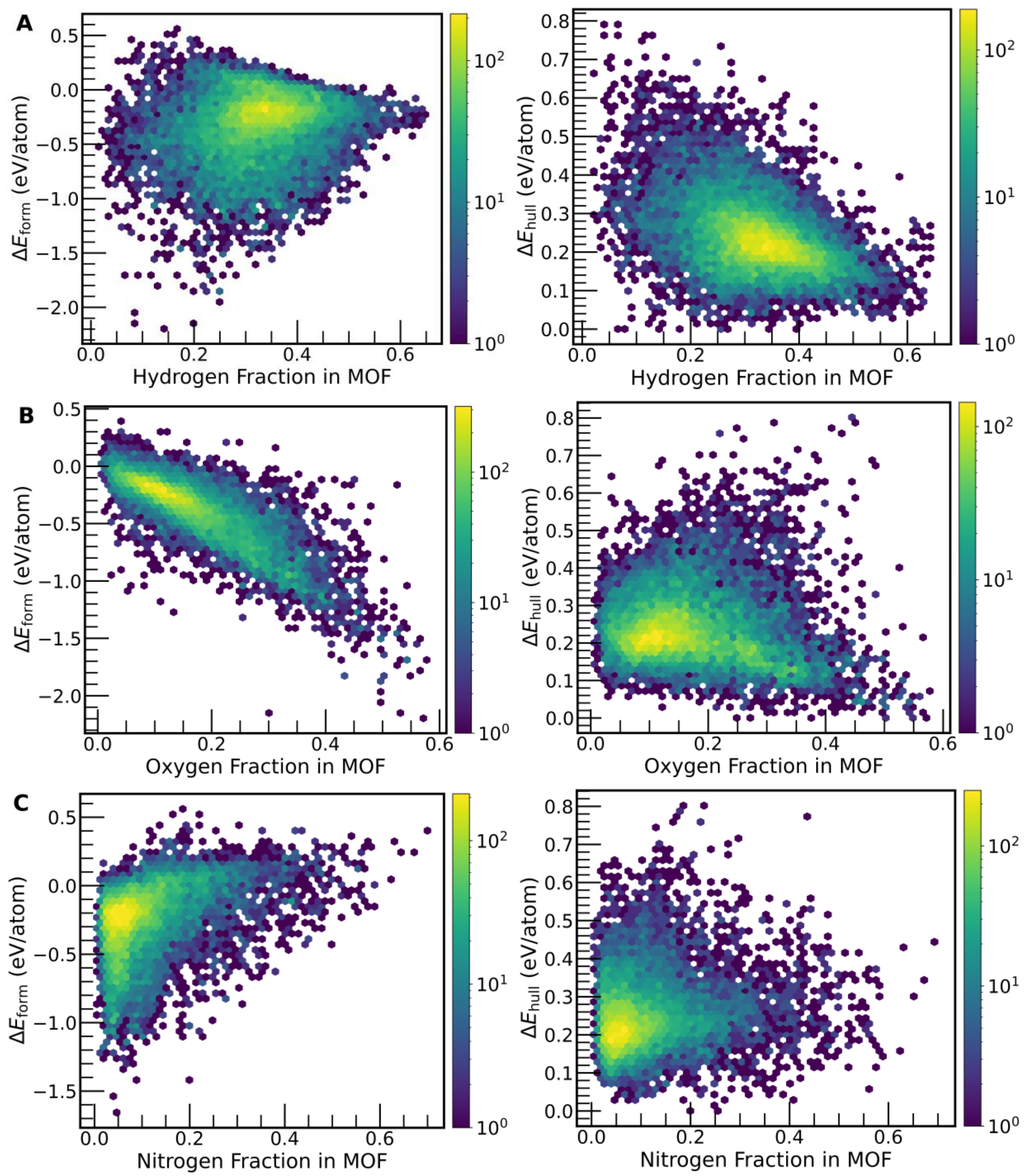

Figure S24. MOF ΔE_{form} and ΔE_{hull} as a function of the stoichiometric (A) hydrogen, (B) oxygen, and (C) nitrogen fraction. Data is for all applicable synthesized and hypothetical MOFs in the QMOF Database. The color bar represents the number of entries in each hexagonal bin.

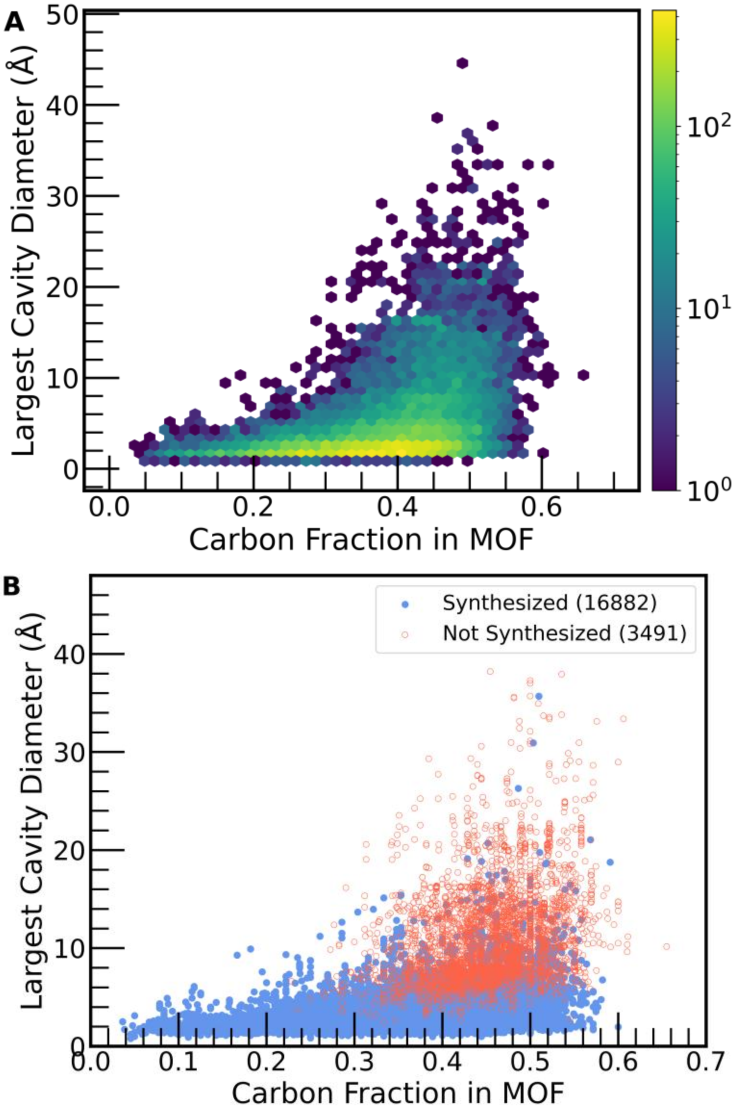

Figure S25. (A, B) MOF largest cavity diameter as a function of the stoichiometric carbon fraction. Data is for all MOFs in the QMOF Database. In (A), the color bar represents the number of entries in each hexagonal bin.

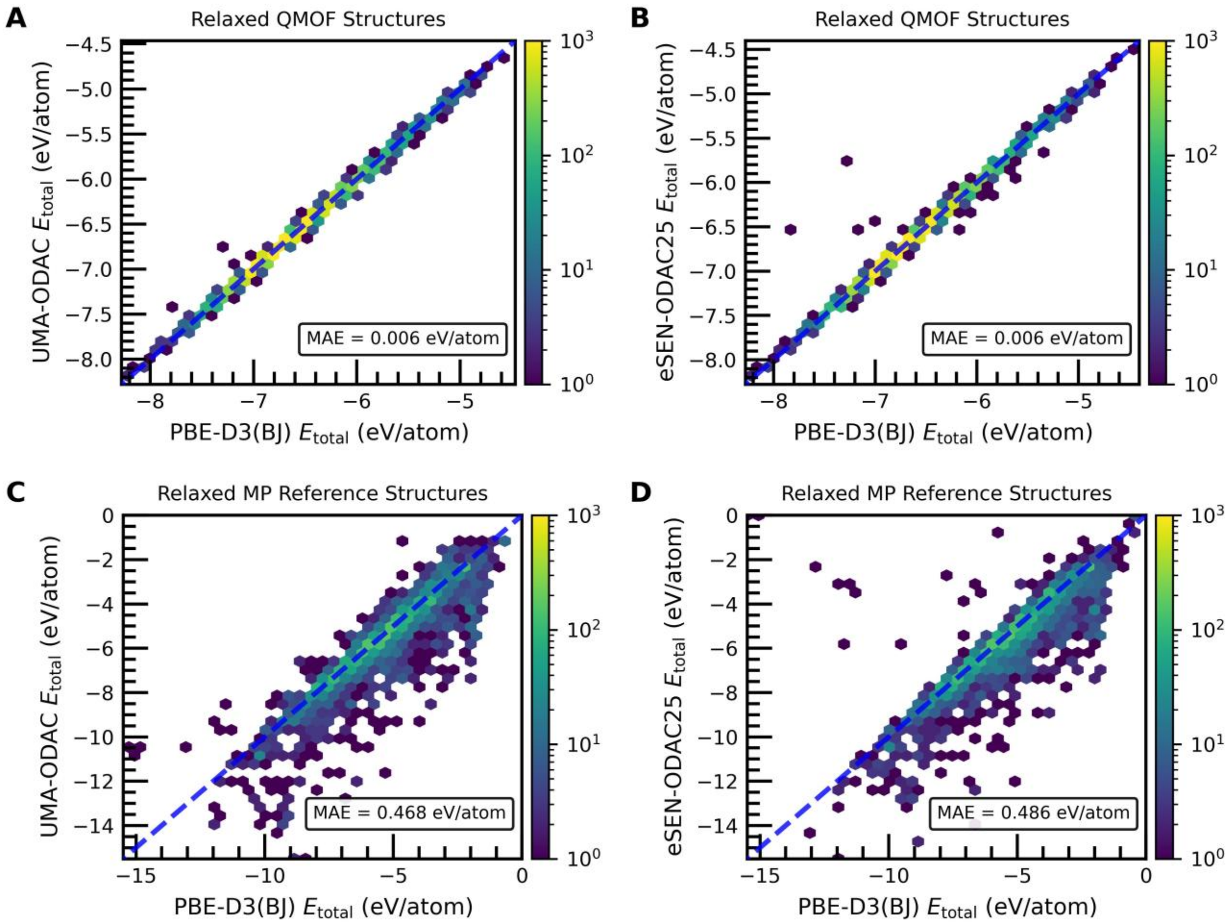

Figure S26. Parity plot of (A) UMA-ODAC and (B) eSEN-ODAC25 versus DFT relaxed total energy in eV/atom of QMOF structures. (C) UMA-ODAC and (D) eSEN-ODAC25 versus DFT relaxed total energy in eV/atom of Materials Project structures. Color bar represents the number of samples in each hexagonal bin. Dashed blue line represents the parity line.

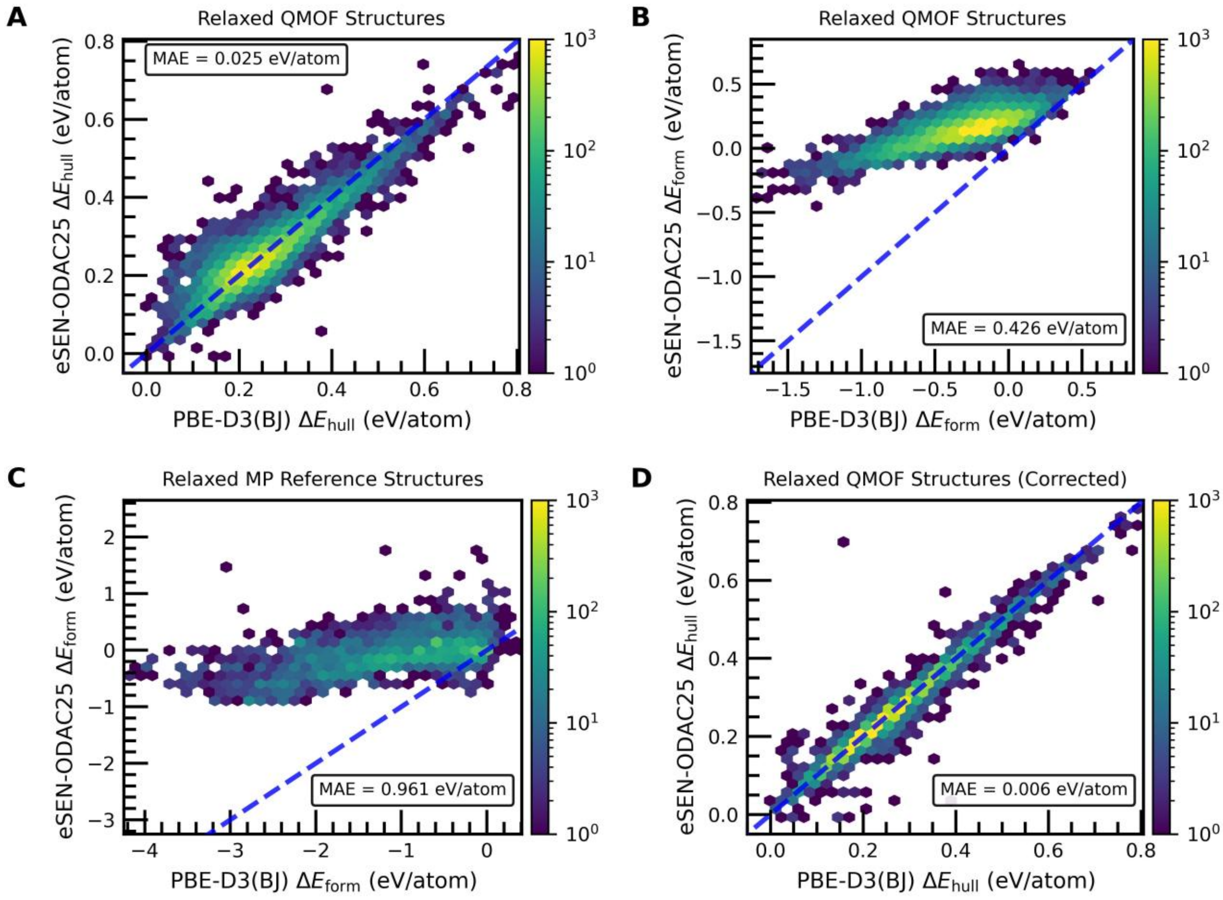

Figure S27. Parity plots for ΔE_{hull}, ΔE_{form}, and total energy using eSEN-ODAC25 and DFT structure relaxations. (A) Parity plot of ΔE_{hull} calculations between eSEN-ODAC25 relaxations and DFT relaxations for the QMOF structures. (B) Parity plot of ΔE_{form} for QMOF and (C) Materials Project (MP) reference structures between eSEN-ODAC25 relaxations and DFT relaxations. (D) Parity plot of ΔE_{hull} calculations using consistent DFT calculated reference structures, between esen-ODAC25 relaxations and DFT relaxations for QMOF structures. The mean absolute error (MAE) is calculated between the eSEN-ODAC25 value and DFT value. The dashed blue line represents the line of parity. The color bar shows the number of entries in each hexagonal bin.

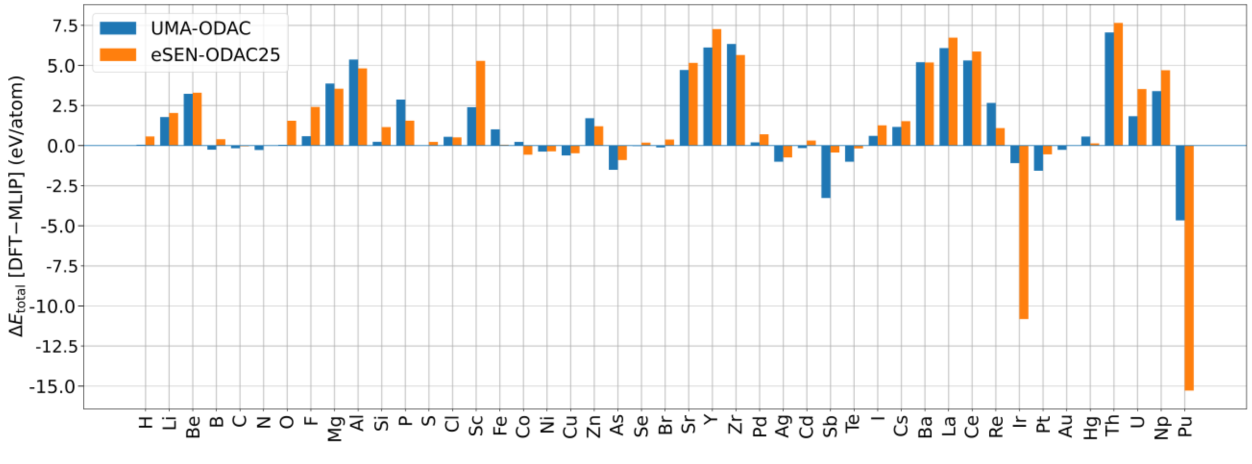

Figure S28. Bar plot of the difference between DFT and MLIP in predicting the total energy for the elemental species. DFT values calculated via PBE-D3(BJ), whereas MLIP values calculated with UMA-ODAC (blue) or eSEN-ODAC25 (orange). The lowest energy elemental structure is shown.

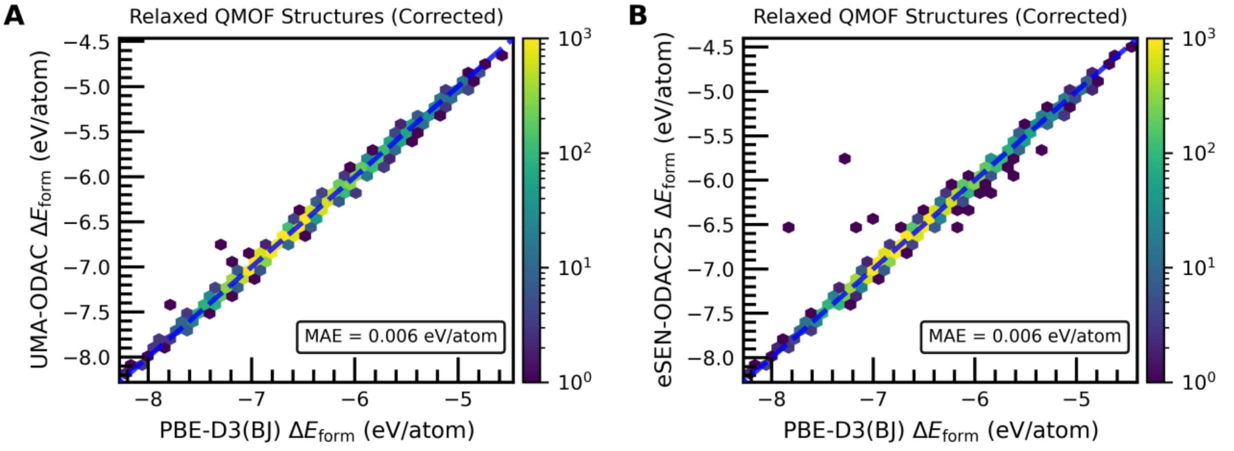

Figure S29. Parity plots comparing ΔE_{form} predicted from MLIP-relaxed versus DFT-relaxed MOF structures. Both calculations of ΔE_{form} use only DFT structure relaxations for the Materials Project elemental reference structures. ΔE_{form} for (A) UMA-ODAC MOF relaxations and (B) eSEN-ODAC25 UMA-ODAC MOF relaxations.

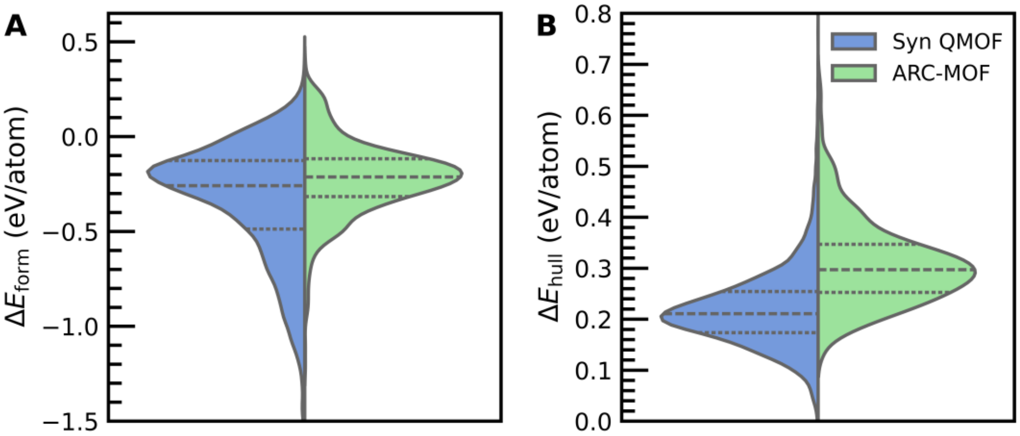

Figure S30. Violin plots of the (A) formation energy (ΔE_{form}) and (B) energy above hull (ΔE_{hull}) for synthesized MOFs (16,882) in the QMOF Database and random hypothetical MOFs (895) from the ARC–MOF Database.[3] The central dashed line represents the median, and the dotted lines represent the first quartile and third quartile in each distribution. ΔE_{form} is defined with respect to elemental phases. The Materials Project reference structures that make up the hull and the synthesized MOFs in the QMOF Database were computed using DFT at the PBE-D3(BJ) level of theory. The hypothetical MOFs from the ARC–MOF Database were computed using the UMA-ODAC MLIP (as in Figure 8D).

Supplementary Methods.

Generative AI MOFs. This section describes the generation, filtering, and validation of diffusion-model generated MOFs using GHP-MOFassemble[4] (commit 39b3d05), MOFDiff[5] v1.0.0, and MOFFUSION[6] (commit 81815bf) in Figure 4. Each model was run using published checkpoints and default configurations. All AI-generated MOF structures were retained only if they contained all or any of C–H–N–O–Zn elements and less than 400 atoms in the primitive unit cell, as determined using Pymatgen. Duplicate structures were detected and removed using Pymatgen's `StructureMatcher` with default settings.

GHP-MOFassemble[4] generates MOF structures using a fixed **pcu** topology through the selection of three different metal nodes. At the fragmentation generation stage, we filtered for fragments containing any or all of C–H–N–O–Zn. In each step of GHP-MOFassemble, we utilized only the Zn paddlewheel and Zn tetramer nodes. Through this process, we generated 30,375 structures. We then filtered our generated structures based on number of atoms, chemical space, and removed duplicates to arrive at a random sample of 100 generated structures. For MOFDiff[5], we utilized the published building block encoder without property optimization to produce coarse-grained MOF structures. After filtering the generated structures, we obtained 131 MOFs. MOFFUSION utilizes multi-modal input to run the diffusion model. For this, we set the text input as the prompt "only C,H,N,O, and Zn. under 400 atoms" and specified to generate 400 structures. We obtained 288 "successful" structures, meaning the structures with cell lengths under 60 Å. After filtering, we retained 38 structures.

With a dataset of AI-generated MOFs, we used the pre-trained machine learning interatomic potentials (MLIPs), TensorNet-MatPES-r^{2}SCAN v2025.1[7,8] and the MatGL 1.2.7[9] library to pre-relax the structures before full DFT relaxation. MLIP relaxations of atomic positions, cell positions, and cell volume occurred until either the maximum force was 0.01 eV/Å or 2500 steps were completed. Most structures reached the step limit without force convergence. To mimic how the generative AI models are likely to be used in practice, after the pre-relaxation with the MLIP, we used MOFChecker[10] v0.9.6 to further filter out MOF structures that did not pass any one of several validity checks. We checked that our structures lack atomic overlaps, over-coordinated or uncoordinated bonds, charged fused rings, or terminal oxygens. We also ensure that our structures are porous. After filtering, 74 out of 100 GHP-MOFassemble structures, 81 out of 131 MOFDiff structures, and 24 out of 38 MOFFUSION structures were retained for subsequent DFT calculations. The remaining AI-generated structures were fully relaxed using DFT with the PBE(D3)-BJ functional as described in the Methods section. Overall, 71 out of 74 GHP-MOFassemble structures, 50 out of 81 MOFDiff structures, and 13 out of 24 MOFFUSION structures were successfully optimized and shown in Figure 4.

Oxidation state determination. To determine the oxidation state of the metal atom in the MOFs, we used oxiMACHINE v0.7.2[11], which is a machine learning model trained on human-labeled oxidation states from the MOF subset of the Cambridge Structural Database.[12] When oxiMACHINE is run on a MOF, the model assigns an oxidation state prediction for each metal atom in the unit cell with a corresponding probability that the assignment is correct. For Figure 6B, MOF entries were only included if they had no mixed oxidation states (e.g. Cu^{+} and Cu^{2+} in a single MOF) and at least one oxidation state assignment with >85% confidence. Using the metal atom identity and the oxidation state, hard/soft/borderline metals were assigned using accepted HSAB classification in the literature.[13–15]

Phonon calculations. Thermal corrections were carried out to determine the Gibbs free energy of formation (ΔG_{form}) and Gibbs free energy above hull (ΔG_{hull}) for 30 random C–H–N–O–Zn MOFs with 150 atoms or less in the QMOF Database using the quasi-harmonic approximation (QHA) as implemented in Phonopy[16,17] v3.1.0 and MatCalc[18] (commit 5545782). To make this task computationally tractable, we adopted the following approximation to correct the 0 K electronic energies from DFT: $G_{\mathrm{DFT}} \approx E_{\mathrm{DFT}} + (G_{\mathrm{MLIP}} - E_{\mathrm{MLIP}})$, where E_{DFT} is the energy of the DFT-optimized structure, E_{MLIP} is the energy of the MLIP-optimized structure, and G_{MLIP} is the MLIP-calculated Gibbs free energy. We used the pre-trained MACE-MP-MOF0-v2 machine learning interatomic potential[19] (MLIP) to describe the MOFs. For the inorganic reference materials that make up the convex hull, structures with a reported ΔE_{hull} between 0 and 0.01 eV/atom on the Materials Project were considered, and the UMA-OMat[20,21] (`uma-s-1p1`) MLIP was adopted. In each case, we relaxed the atomic positions, unit cell shape, and volume until the magnitude of the forces on all atoms was below 10^{-6} eV/Å. Then, ±3% linear strain in increments of 1% was applied to the unit cell after which each structure was relaxed using the aforementioned settings but at fixed volume, while allowing the atomic positions and unit cell shape to change. For the subsequent phonon calculations, supercells of the converged structures were created such that the cell lengths were at least 20 Å in each dimension. If imaginary modes were present following the phonon calculations (defined here as a frequency

less than –0.1 THz), we randomly rattled the atoms using a standard deviation of 0.01 Å as implemented in ASE, after which the structures were re-relaxed. The Gibbs free energy was then determined at various temperatures and a constant pressure of 1 bar under the QHA limit. To properly account for significant entropic effects from gas-phase species, we treated the following decomposition products in the C–H–N–O–Zn chemical space as gases rather than crystalline solids and used ideal gas thermochemistry: H_2, O_2, CH_4, CO_2, CO, NO, NO_2, N_2, H_2O, NH_3, HCN, and H_2CO. These gases were modeled as a single molecule in the center of a periodic box with 20 Å of vacuum in each dimension and relaxed at fixed cell volume using the methods outlined in Methods–Density Functional Theory to get the DFT-based 0 K energy. The molecules were then re-relaxed using the UMA-OMol[20,22] (`uma-s-1p1`) MLIP via MatCalc, and the vibrational modes were calculated using ASE's Vibrations class. This data was used to determine the Gibbs free energy correction to the 0 K energy from DFT via ASE's `IdealGasThermo` class. The resulting Gibbs free formation energies and Gibbs free energies above hull are given in Figure S6.

Miscellaneous. To ensure matching pseudopotentials between our DFT calculations and the pre-trained MLIPs, structures containing any of the following elements were removed: Bi, Ca, Cr, Dy, Er, Eu, Ga, Gd, Ge, Hf, Ho, In, K, Lu, Mn, Mo, Na, Nb, Nd, Pb, Pm, Po, Pr, Rb, Rh, Ru, Sm, Sn, Ta, Tb, Tc, Ti, Tl, Tm, V, W, Yb. VESTA[23] was used for structure visualization and figure creation in Figure S17. Ionic radii used in Figure S19 were obtained from the Shannon ionic radii database[24] via Pymatgen[25] v. 2025.1.9. To determine the linker identities used in Figure 6C, S20, and S21, linker formulas were first extracted using MOFid[1] then functional groups were identified using RDKit[26] v2025.03.6.

References.

(1) Bucior, B. J.; Rosen, A. S.; Haranczyk, M.; Yao, Z.; Ziebel, M. E.; Farha, O. K.; Hupp, J. T.; Siepmann, J. I.; Aspuru-Guzik, A.; Snurr, R. Q. Identification Schemes for Metal–Organic Frameworks To Enable Rapid Search and Cheminformatics Analysis. *Cryst. Growth Des.* **2019**, *19* (11), 6682–6697. https://doi.org/10.1021/acs.cgd.9b01050.

(2) Zhao, G.; Brabson, L. M.; Chheda, S.; Huang, J.; Kim, H.; Liu, K.; Mochida, K.; Pham, T. D.; Prerna; Terrones, G. G.; Yoon, S.; Zoubritzky, L.; Coudert, F.-X.; Haranczyk, M.; Kulik, H. J.; Moosavi, S. M.; Sholl, D. S.; Siepmann, J. I.; Snurr, R. Q.; Chung, Y. G. CoRE MOF DB: A Curated Experimental Metal-Organic Framework Database with Machine-Learned Properties for Integrated Material-Process Screening. *Matter* **2025**, *8* (6). https://doi.org/10.1016/j.matt.2025.102140.

(3) Burner, J.; Luo, J.; White, A.; Mirmiran, A.; Kwon, O.; Boyd, P. G.; Maley, S.; Gibaldi, M.; Simrod, S.; Ogden, V.; Woo, T. K. ARC–MOF: A Diverse Database of Metal-Organic Frameworks with DFT-Derived Partial Atomic Charges and Descriptors for Machine Learning. *Chem. Mater.* **2023**, *35* (3), 900–916. https://doi.org/10.1021/acs.chemmater.2c02485.

(4) Park, H.; Yan, X.; Zhu, R.; Huerta, E. A.; Chaudhuri, S.; Cooper, D.; Foster, I.; Tajkhorshid, E. A Generative Artificial Intelligence Framework Based on a Molecular Diffusion Model for the Design of Metal-Organic Frameworks for Carbon Capture. *Commun. Chem.* **2024**, *7* (1), 21. https://doi.org/10.1038/s42004-023-01090-2.

(5) Fu, X.; Xie, T.; Rosen, A. S.; Jaakkola, T.; Smith, J. MOFDiff: Coarse-Grained Diffusion for Metal-Organic Framework Design. arXiv 2023. https://doi.org/10.48550/ARXIV.2310.10732.

(6) Park, J.; Lee, Y.; Kim, J. Multi-Modal Conditional Diffusion Model Using Signed Distance Functions for Metal-Organic Frameworks Generation. *Nat. Commun.* **2025**, *16* (1), 34. https://doi.org/10.1038/s41467-024-55390-9.

(7) Kaplan, A. D.; Liu, R.; Qi, J.; Ko, T. W.; Deng, B.; Riebesell, J.; Ceder, G.; Persson, K. A.; Ong, S. P. A Foundational Potential Energy Surface Dataset for Materials. arXiv March 6, 2025. https://doi.org/10.48550/arXiv.2503.04070.

(8) Simeon, G.; De Fabritiis, G. TensorNet: Cartesian Tensor Representations for Efficient Learning of Molecular Potentials. *Adv. Neural Inf. Process. Syst.* **2023**, *36*, 37334–37353.

(9) Ko, T. W.; Deng, B.; Nassar, M.; Barroso-Luque, L.; Liu, R.; Qi, J.; Thakur, A. C.; Mishra, A. R.; Liu, E.; Ceder, G.; Miret, S.; Ong, S. P. Materials Graph Library (MatGL), an Open-Source Graph Deep

Learning Library for Materials Science and Chemistry. *Npj Comput. Mater.* **2025**, *11* (1), 253. https://doi.org/10.1038/s41524-025-01742-y.
(10) Jin, X.; Jablonka, K. M.; Moubarak, E.; Li, Y.; Smit, B. MOFChecker: A Package for Validating and Correcting Metal–Organic Framework (MOF) Structures. *Digit. Discov.* **2025**, *4* (6), 1560–1569. https://doi.org/10.1039/D5DD00109A.
(11) Jablonka, K. M.; Ongari, D.; Moosavi, S. M.; Smit, B. Using Collective Knowledge to Assign Oxidation States of Metal Cations in Metal–Organic Frameworks. *Nat. Chem.* **2021**, *13* (8), 771–777. https://doi.org/10.1038/s41557-021-00717-y.
(12) Moghadam, P. Z.; Li, A.; Wiggin, S. B.; Tao, A.; Maloney, A. G. P.; Wood, P. A.; Ward, S. C.; Fairen-Jimenez, D. Development of a Cambridge Structural Database Subset: A Collection of Metal–Organic Frameworks for Past, Present, and Future. *Chem. Mater.* **2017**, *29* (7), 2618–2625. https://doi.org/10.1021/acs.chemmater.7b00441.
(13) Pearson, R. G. Hard and Soft Acids and Bases. *J. Am. Chem. Soc.* **1963**, *85* (22), 3533–3539. https://doi.org/10.1021/ja00905a001.
(14) Hamisu, A. M.; Ariffin, A.; Wibowo, A. C. Cation Exchange in Metal-Organic Frameworks (MOFs): The Hard-Soft Acid-Base (HSAB) Principle Appraisal. *Inorganica Chim. Acta* **2020**, *511*, 119801. https://doi.org/10.1016/j.ica.2020.119801.
(15) Pearson, R. G. Hard and Soft Acids and Bases, HSAB, Part 1: Fundamental Principles. *J. Chem. Educ.* **1968**, *45* (9), 581. https://doi.org/10.1021/ed045p581.
(16) Togo, A. First-Principles Phonon Calculations with Phonopy and Phono3py. *J. Phys. Soc. Jpn.* **2023**, *92* (1), 012001. https://doi.org/10.7566/JPSJ.92.012001.
(17) Togo, A.; Chaput, L.; Tadano, T.; Tanaka, I. Implementation Strategies in Phonopy and Phono3py. *J. Phys. Condens. Matter* **2023**, *35* (35), 353001. https://doi.org/10.1088/1361-648X/acd831.
(18) Kozeschnik, E. Mean-Field Microstructure Kinetics Modeling. In *Encyclopedia of Materials: Metals and Alloys*; Elsevier, 2022; pp 521–526. https://doi.org/10.1016/B978-0-12-819726-4.00055-7.
(19) Elena, A. M.; Kamath, P. D.; Jaffrelot Inizan, T.; Rosen, A. S.; Zanca, F.; Persson, K. A. Machine Learned Potential for High-Throughput Phonon Calculations of Metal—Organic Frameworks. *Npj Comput. Mater.* **2025**, *11* (1), 125. https://doi.org/10.1038/s41524-025-01611-8.
(20) Wood, B. M.; Dzamba, M.; Fu, X.; Gao, M.; Shuaibi, M.; Barroso-Luque, L.; Abdelmaqsoud, K.; Gharakhanyan, V.; Kitchin, J. R.; Levine, D. S.; Michel, K.; Sriram, A.; Cohen, T.; Das, A.; Rizvi, A.; Sahoo, S. J.; Ulissi, Z. W.; Zitnick, C. L. UMA: A Family of Universal Models for Atoms. arXiv June 30, 2025. https://doi.org/10.48550/arXiv.2506.23971.
(21) Barroso-Luque, L.; Shuaibi, M.; Fu, X.; Wood, B. M.; Dzamba, M.; Gao, M.; Rizvi, A.; Zitnick, C. L.; Ulissi, Z. W. Open Materials 2024 (OMat24) Inorganic Materials Dataset and Models. arXiv October 16, 2024. http://arxiv.org/abs/2410.12771 (accessed 2024-10-17).
(22) Levine, D. S.; Shuaibi, M.; Spotte-Smith, E. W. C.; Taylor, M. G.; Hasyim, M. R.; Michel, K.; Batatia, I.; Csányi, G.; Dzamba, M.; Eastman, P.; Frey, N. C.; Fu, X.; Gharakhanyan, V.; Krishnapriyan, A. S.; Rackers, J. A.; Raja, S.; Rizvi, A.; Rosen, A. S.; Ulissi, Z.; Vargas, S.; Zitnick, C. L.; Blau, S. M.; Wood, B. M. The Open Molecules 2025 (OMol25) Dataset, Evaluations, and Models. arXiv May 13, 2025. https://doi.org/10.48550/arXiv.2505.08762.
(23) Momma, K.; Izumi, F. *VESTA 3* for Three-Dimensional Visualization of Crystal, Volumetric and Morphology Data. *J. Appl. Crystallogr.* **2011**, *44* (6), 1272–1276. https://doi.org/10.1107/S0021889811038970.
(24) Shannon, R. D. Revised Effective Ionic Radii and Systematic Studies of Interatomic Distances in Halides and Chalcogenides. *Acta Crystallogr. Sect. A* **1976**, *32* (5), 751–767. https://doi.org/10.1107/S0567739476001551.
(25) Ong, S. P.; Richards, W. D.; Jain, A.; Hautier, G.; Kocher, M.; Cholia, S.; Gunter, D.; Chevrier, V. L.; Persson, K. A.; Ceder, G. Python Materials Genomics (Pymatgen): A Robust, Open-Source Python Library for Materials Analysis. *Comput. Mater. Sci.* **2013**, *68*, 314–319.
(26) Greg Landrum; Paolo Tosco; Brian Kelley; Ricardo Rodriguez; David Cosgrove; Riccardo Vianello; sriniker; Peter Gedeck; Gareth Jones; Eisuke Kawashima; NadineSchneider; Dan Nealschneider;

Andrew Dalke; tadhurst-cdd; Matt Swain; Brian Cole; Samo Turk; Aleksandr Savelev; Alain Vaucher; Maciej Wójcikowski; Ichiru Take; Hussein Faara; Rachel Walker; Vincent F. Scalfani; Daniel Probst; Kazuya Ujihara; Niels Maeder; Axel Pahl; guillaume godin; Juuso Lehtivarjo. Rdkit/Rdkit: 2025_03_6 (Q1 2025) Release, 2025. https://doi.org/10.5281/ZENODO.16996017.
